# Efficacy and safety of statins, ezetimibe and statins-ezetimibe therapies for children and adolescents with heterozygous familial hypercholesterolaemia: Systematic review, pairwise and network meta-analyses of randomised controlled trials

**DOI:** 10.1016/j.atherosclerosis.2024.118598

**Published:** 2025-02

**Authors:** Alexis Llewellyn, Mark Simmonds, David Marshall, Melissa Harden, Beth Woods, Steve E. Humphries, Uma Ramaswami, Lorraine Priestley-Barnham, Mark Fisher, Laila J. Tata, Nadeem Qureshi

**Affiliations:** a Centre for Reviews and Dissemination, University of York, UK; b Centre for Health Economics, University of York, UK; c Centre for Cardiovascular Genetics, University College London, UK; d Royal Free Hospital and Genetics and Genomic Medicine, University College London, UK; e Royal Brompton and Harefield Hospital, Guy's and St Thomas NHS Trust, UK; f Lifespan and Population Health Unit and Centre for Perinatal Research, School of Medicine, University of Nottingham, UK; g NIHR School of Primary Care Research, University of Nottingham, UK

**Keywords:** Statins, Ezetimibe, Children, Adolescents, Familial hypercholesterolaemia, Systematic review, Network meta-analysis

## Abstract

**Background and aims:**

Statins, ezetimibe and statins-ezetimibe combination therapy are recommended lipid-lowering therapies (LLTs) in children with heterozygous familial hypercholesterolaemia (HeFH). However, their relative effectiveness is not well understood. We aimed to compare the safety and efficacy of these therapies using direct and indirect comparisons.

**Methods:**

We conducted systematic review, pairwise and network meta-analyses (NMAs) of randomised-controlled trials (RCTs) of statins, ezetimibe and statins-ezetimibe combination therapy in people <18 years with HeFH. Comprehensive bibliographic searches were conducted in December 2022, and a Medline update in January 2024. NMA models accounted for drug class, statin type and dosage.

**Results:**

Thirteen RCTs were included (n = 1649, median age 13 years, follow-up 6 weeks-2 years). All LLTs reduced low-density lipoprotein cholesterol (LDL-C) and total cholesterol; statins led to increases in high-density lipoprotein cholesterol and reductions in triglycerides. Statins reduced LDL-C by 33.61 % against placebo (95 % CI 27.58 to 39.63, I^2^ = 83 %). Adding ezetimibe to statins reduced LDL-C by an additional 15.85 % (95 % CI 11.91 to 19.79). NMAs showed intermediate-dose statins reduced LDL-C by an additional 4.77 % compared with lower-doses statins (95 % CrI −11.22 to 1.05); higher-dose statins and intermediate-dose statins + ezetimibe may be similarly effective and are probably superior to ezetimibe, intermediate-and lower-dose statins. There was no evidence of differences in maturation, safety or tolerability between LLTs and placebo.

**Conclusions:**

Statins, ezetimibe and statins-ezetimibe are all effective treatments for children with HeFH, but the magnitude of LDL-C reductions varies and may depend on treatment dosage and combination. No safety or tolerability issues were found. Longer-term safety and effectiveness are uncertain.

## Highlights

• 1st network meta-analysis of lipid lowering therapies (LLTs) for children with familial hypercholesterolaemia.
• All LLTs led to clinically significant reductions in LDL cholesterol (LDL-C) and were generally well tolerated.
• Statins dosage and combination with ezetimibe are likely to affect the magnitude of LDL-C reductions.
• Higher-dose statins and intermediate-dose statins + ezetimibe are likely superior to intermediate and lower dose therapies.
• Intermediate-dose statins may be slightly more effective than lower-dose statins, although differences are probably small.

## Graphical abstract

**Figure:**
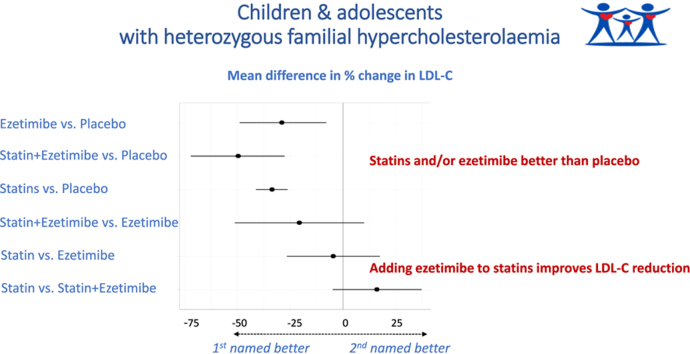

## 1 Introduction

Familial hypercholesterolaemia (FH) is one of the most common inherited metabolic diseases. Approximately 1 in 250 individuals are affected by its heterozygous form (HeFH) worldwide [1]. FH is characterised by elevated low-density lipoprotein cholesterol (LDL-C) concentrations. Individuals with HeFH have a lifelong elevation of serum LDL cholesterol that is two-to three times higher than people without FH. Early atherosclerosis can be detected in untreated FH from the second decade of life [3], and HeFH increases the risk of premature cardiovascular disease (CVD) as early as people's mid-thirties [4]. For individuals with FH, the duration of exposure to high LDL-C is a key determinant of CVD risk, also called “cholesterol burden” [5,6].

Statins are the primary treatment for children with FH, and atorvastatin, lovastatin, pravastatin, rosuvastatin and simvastatin are used in children internationally. European guidelines for children with FH recommend that statin treatment should be considered early (typically between 8 and 10 years, although as early as 6 years depending on specific risk factors). Statin treatment should be started with low doses and increased to reach treatment goals, which vary by age; LDL-C should be lowered by 50 % children younger than 10 years, and to below 3.5 mmol/l in children over 10 years [2,7]. However, dose-intensity classification is not clearly defined in children; in adults, it is based on expected percentage LDL-C reductions (≥50 % for high-intensity, 30 to <50 % for moderate-intensity and <30 % for low-intensity dosage) [8].

Addition of ezetimibe, a cholesterol absorption inhibitor that lowers LDL-C and other key lipid/lipoprotein variables, is also recommended to attain LDL-C targets [2,9,10]. In case of intolerance to statins, ezetimibe monotherapy has been recommended [2]. Newer generation PCSK-9 inhibitor therapies (alirocumab, evolocumab) have been licensed but are not currently included in guidelines for children with FH [11,12].

Evidence for the relative effectiveness and safety of statins monotherapy, ezetimibe monotherapy and combination therapy with statins and ezetimibe in children with HeFH is limited. Whilst there is substantive short-term evidence from systematic reviews of randomised controlled trials (RCTs) comparing statins with placebo for reducing LDL-C in HeFH children [[13], [14], [15]], evidence evaluating ezetimibe monotherapy or statins-ezetimibe combination therapy is more limited, and there is no head-to-head RCT evidence comparing the relative effectiveness and safety of ezetimibe monotherapy against statins alone or statins/ezetimibe combination therapy [16,17]. There is also no head-to-head RCT evidence comparing the relative benefits and safety of different statins for children with HeFH. There is no meta-analysis comparing the totality of the RCT evidence for statins, ezetimibe monotherapy and statins/ezetimibe combination therapy in children with FH. Previous systematic reviews only included placebo-controlled trials of statins and excluded head-to-head comparisons of active treatments and trials evaluating ezetimibe with and without statins. Significant heterogeneity was found in LDL-C outcomes, but sources of heterogeneity (such as statins dosage) were either not assessed, or only explored using standard subgroup analyses, which have known limitations [[13], [14], [15]]. The last review on the topic was published in 2020 [14].

Network meta-analyses permit the comparison of the totality of evidence for a group of interventions, by providing relative effect estimates of all interventions compared with every other using both direct and indirect evidence, incorporating effects from trials with more than two arms appropriately (accounting for double counting), and ranking all the interventions coherently [18]. This review aims to pool the totality of the RCT evidence on the main recommended LLT pharmacotherapies in children with HeFH, and to formally assess potential sources of heterogeneity (including LLT type and dosage) using advanced meta-analytic techniques. We present a systematic review and meta-analysis with network meta-analysis of all published randomised controlled trials (RCTs) comparing statins, ezetimibe and statin-ezetimibe combination therapy in children with HeFH. The project was funded by the National Institute for Health and Care Research [19].

## 2 Materials and methods

This review was conducted following the Centre for Reviews and Dissemination guidance on undertaking systematic reviews and reported following the principles of the PRISMA statement [20,21], and is registered on PROSPERO. [CRD42023408037] [22].

### 2.1 Bibliographic searches and study selection

Comprehensive searches were undertaken in December 2022 to identify published and unpublished studies of statins in children with HeFH. MEDLINE (Ovid), Embase (Ovid), CENTRAL (Cochrane Library), Cochrane Database of Systematic Reviews (Cochrane Library), Science Citation Index (Web of Science) and the International Health Technology Assessment (INAHTA) database were searched, along with searches of trial registries, sources of unpublished and ongoing studies, guideline resources and reference checking of systematic reviews and included studies (Supplementary file 1). An update search of MEDLINE was performed in January 2024. Two researchers (AL, DM) independently screened all titles and abstracts and all papers retrieved for full text examination to determine inclusion following pre-specified criteria. Disagreements were resolved through discussion.

### 2.2 Selection criteria

RCTs of children and young people up to age 18 years with a clinical diagnosis of HeFH, according to genetic testing, family history and/or serum lipid profile with symptoms consistent with HeFH were included. Studies including mixed populations (e.g. dyslipidaemia and FH) that reported results for HeFH individuals separately were included. Homozygous FH studies were excluded. Randomised trials of any statins or ezetimibe, alone or in combination, compared to each another (including different doses of the same statin), to placebo or to diet alone were included. Other therapies including PCSK-9 inhibitors, resins, fibrates, and non-pharmacological therapies were excluded. Eligible outcomes included changes in serum LDL-C (e.g. mean change from baseline, achievement of target LDL-C), serum total cholesterol, serum HDL-C and triglyceride levels; carotid intima-media thickness (CIMT), and changes in endothelial function. Change in measures of growth and maturation (e.g. increase in Tanner stage), adverse events, including myopathy, liver dysfunction, rhabdomyolysis and discontinuation due to adverse event were also eligible.

### 2.3 Data extraction and risk of bias assessment

Data on study design and intervention characteristics (including statin dose and type) and participants characteristics (including age, sex, LDL-C at baseline) and eligible outcomes were extracted from included studies by one reviewer and checked by a second (AL, DM). Percentage change and absolute change in cholesterol levels from baseline were calculated for each included trial from the data presented for all trial arms and at all reported follow-up times. To inform analyses accounting for dosage, LLT were coded according to dosage (lower, intermediate, higher doses). In the absence of paediatric standards, coding was informed by guidelines for adults and meta-analytic evidence on dose-intensity [8,[23], [24], [25]]. Information on whether trials reported any patient and public involvement or engagement (PPIE) input was sought but not found. The classification is presented in Supplementary file 2. Risk of bias was assessed by one reviewer and checked by a second using the Cochrane RoB 2 tool [26].

### 2.4 Synthesis

#### 2.4.1 Pairwise meta-analyses

Where at least two studies reported a given outcome, effect estimates were pooled across studies using standard random and fixed effects meta-analysis and results presented in forest plots. Continuous outcomes (percentage and absolute reduction from baseline in cholesterol) were analysed in terms of mean differences (MDs) between arms; dichotomous outcomes (e.g. adverse events) were analysed using relative risks. Heterogeneity was assessed in terms of I^2^ and by inspecting the between-study heterogeneity standard deviation (τ) relative to the treatment effect size. Separate meta-analyses were conducted for each lipid-lowering medication (statins only, ezetimibe only, and combination of statins and ezetimibe). Where more than one follow-up time was reported for the same treatment and trial arm, the last follow-up value (within the blinded period) was used. Where trials used multiple doses of the treatment, only the highest dose was included (except for analyses split by dosage). The relative effectiveness of different statin types and doses, and between statins and ezetimibe used alone and in combination was explored using standard subgroup analyses. Meta-analyses were conducted in R using the publicly available meta library. Publication bias was assessed using standard methods [27,28].

#### 2.4.2 Network meta-analyses

NMA is an extension of pairwise (two-treatment) meta-analysis that allows comparisons across three or more treatments by producing relative effects for every pair of treatments in a connected network. Outcomes data from studies comparing interventions directly is pooled with indirect evidence from studies with a common comparator. This allows for consistent estimates of relative effects accounting for all relevant evidence [18]. Random effects NMAs were conducted with a Bayesian framework and Markov Chain Monte Carlo methods using the multinma library in R where there were sufficient data. Relative percentage changes in LDL-C were considered in three network models: the first treated all statins as equivalent (but different from ezetimibe and statins-ezetimibe combination therapy); the second treated each statin (and ezetimibe with/without statins) as a different treatment; the third treated each dose level (higher, intermediate or lower dose) of statin as a different treatment.

#### 2.4.3 Narrative synthesis

Where fewer than two studies were identified for a given outcome, or where data were too sparse for pooling, results were reported narratively and tabulated following appropriate guidance [29].

## 3 Results

### 3.1 Study and participant characteristics

The study selection process is reported in Supplementary Fig. 1. Thirteen unique trials were included, for a total of 1649 children and adolescents [3,16,17,[30], [31], [32], [33], [34], [35], [36], [37], [38], [39]].

Table 1 summarises study characteristics. Nine placebo-controlled trials of statins [3,[30], [31], [32], [33], [34],^36^,^38^,^39^], two head-to-head comparisons between different doses of the same statin only (pitavastatin 1–2 mg) and lovastatin (10–40 mg) [35,37], one placebo-controlled trial of ezetimibe monotherapy (10 mg) [16], and one trial comparing ezetimibe (10 mg)-simvastatin (10–40 mg) combination therapy against placebo and simvastatin (10–40 mg) were included [17]. Three trials evaluated lovastatin (10–40 mg) [32,37,39] two evaluated pitavastatin (1–4 mg) [31,35], pravastatin (5–40 mg) [3,36] and simvastatin (10–40 mg) respectively, [33,34]. Atorvastatin (10–20 mg) [38] and rosuvastatin (5–20 mg) [30] were each evaluated in a single trial. Ten trials were conducted in more than one centre [16,17,[30], [31], [32],^34^,^35^,[37], [38], [39]]. All used a parallel-group design with a run-in phase including a fat-restricted diet ranging between four weeks to three months. Five trials included statin dose escalation [17,32,34,38,39]. Sample size varied from 14 to 248, and median follow-up duration was 24 weeks (range six weeks to two years).

**Table 1:** Characteristics of included studies.

	Multi-centre	N randomised	N arms	Intervention	Doses (mg/day)	Follow-up (wks)	Age (mean)	Male (%)	Baseline LDL-C (mean, mmol/L; md/dL)
***Statins vs placebo***									
Avis 2010 [30]	Yes	177	4	Rosuvastatin	5, 10, 20	12	14.5	55	6.0; 233
Braamskamp 2015 [31]	Yes	106	4	Pitavastatin	1, 2, 4	12	10.6	45	6.0; 232
Clauss 2005 [32]	Yes	54	2	Lovastatin	20, then 40^a^	24	15	0	5.6; 215
Couture 1998 [33]	No	63	2	Simvastatin	20	6	12.6	59	5.8; 224
de Jongh 2002 [34]	Yes	175	2	Simvastatin	10, then 20, then 40^b^	48	14.2	57	5.4; 209
Knipscheer 1996 [36]	NR	72	4	Pravastatin	5, 10, 20	12	12	35	6.5; 251
McCrindle 2003 [38]	Yes	187	2	Atorvastatin	10, then 10 to 20^c^	26	14.1	69	5.7; 220
Stein 1999 [39]	Yes	132	2	Lovastatin	10, then 20, then 40^b^	48	13.2	100	6.5; 251
Wiegman 2004 [3]	No	214	2	Pravastatin	20, 40^d^	104	13	47	6.2; 240
***Statinsvs.statins (dose comparisons)***									
Harada-Shiba 2016 [35]	Yes	14	2	Pitavastatin	1 or 2	52	11.8	100	6.8; 262
Lambert 1996 [37]	Yes	69	4	Lovastatin	10 or 20 or 30 or 40	8	12.9	NR	6.2; 242
***Ezetimibevs.placebo***									
Kusters 2015 [16]	Yes	138	2	Ezetimibe	10	12	8.3	43	5.9; 228
***Ezetimibe + statinsvs.placebo + statins***									
Van der Graff 2008 [17]	Yes	248	6	Ezetimibe-+Simvastatin	Ez: 10; Sta: 10 or 20 or 40, then 40^e^	33	14.1	57	5.8; 225

a 20 mg/day up to week 4.

b 10 mg/day up to 8 weeks, then 20 mg/day up to 16 weeks.

c 20 mg/day if LDL-C levels >3.4 mmol/L (130 mg/dL) at week 4.

d 20 mg/day for <14 years, 40 mg/day for ≥14 years.

e Simvastatin 10/20, or 40 mg/day + ezetimibe 10 mg/day or placebo for 6 weeks; then simvastatin 40 mg/day + ezetimibe 10 mg/day or placebo for 27 weeks.

Baseline participant characteristics are summarised in Table 1, and trial selection criteria in Supplementary Table 1. Participant mean age ranged from 8.3 to 15 years. Two studies only included boys [35,39] and one only trial included post-menarchal girls [32]. Most participants were white (80 %–96 %). All trials included children with a diagnosis of HeFH exclusively, except one which included a 10 % subset of non-FH patients with LDL-C above 4.13 mmol/l (160 mg/dL) that was stratified at randomisation [16]. Mean baseline LDL-C ranged from 5.4 mmol/L (209 mg/dL) to 6.8 mmol/L (262 mg/dL). Average BMI was within a healthy range where reported (18.7–23 kg/m^2^).

### 3.2 Risk of bias assessment

For a full risk of bias assessment see Supplementary Table 2. Two studies were at low risk of bias overall [16,32], none were considered high risk, and 11 raised some concerns [3,17,30,31,[33], [34], [35], [36], [37], [38], [39]], primarily due to insufficient reporting of the randomisation process. Study reporting was insufficient to assess selective outcome reporting in all studies.

### 3.3 Clinical effectiveness

#### 3.3.1 LDL-C

##### 3.3.1.1 Pairwise comparisons

**Fig. 1:**
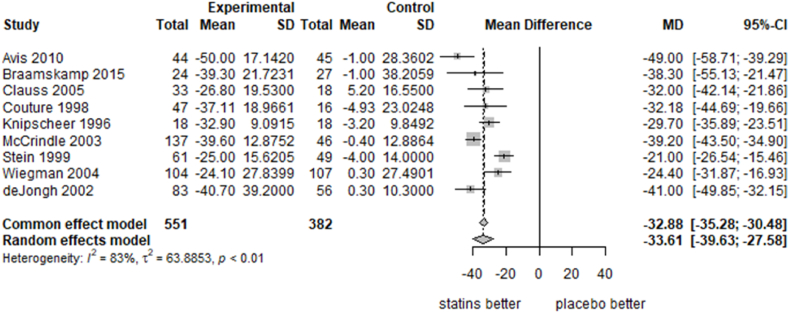
Forest plot, statins *vs.* placebo, % change from baseline in serum LDL-C at end of follow-up.

Fig. 1 shows the pairwise meta-analysis of the impact of statins on serum LDL-C compared to placebo. Statins reduced LDL-C by 33.61 % more than placebo (95 % CI 27.58 to 39.63, I^2^ = 83 %, 9 trials). This equates to a 2.02 mmol/L reduction in LDL-C (95 % CI 1.63 to 2.40, I^2^ = 69 %, 9 trials) (see Supplementary Fig. 2). There was no evidence of publication bias (Begg test p = 0.532), although tests are limited by study numbers (Supplementary Fig. 19).

Subgroup analyses (Supplementary Figs. 3–6) showed some evidence that the effectiveness of statins varies with statin dosage, with higher doses leading to greater reductions in LDL-C. This may explain some of the heterogeneity observed in Fig. 1. There was some evidence that different statins may have differing effectiveness, but the small numbers of trials, and confounding with dosage makes it difficult to draw any firm conclusions. There was no evidence that the effectiveness of statins varied with trial duration or LDL-C at baseline.

Ezetimibe reduced LDL-C by −28.95 % (95 % CI -32.10 to −25.80) and −1.63 mg/dL (95 % CI, −2.22 to −1.04, 1 trial) against placebo. Compared with simvastatin alone, the addition of ezetimibe reduced LDL-C by an additional −15.85 % (95 % CI -19.79 to −11.91) and −0.97 mg/dL (95 % CI -1.36 to −0.58, 1 trial). Pairwise comparisons are summarised in Supplementary Table 3.

Percentages of participants achieving an LDL-C target (<3.4 mmol/L or <2.8 mm/L) were reported by three trials and could not be pooled due to insufficient data [17,30,38]; these are summarised in Supplementary Table 4. The <3.4 mmol/L target was achieved by between 53 % and 60 % of participants on statins [17,38], and by 77 % of participants with ezetimibe-statins combination [17]. Between 12 % and 41 % reached the <2.8 mmol/L target on statins [30], and 63 % with the addition of ezetimibe [17].

##### 3.3.1.2 NMA networks summary

**Fig. 2:**
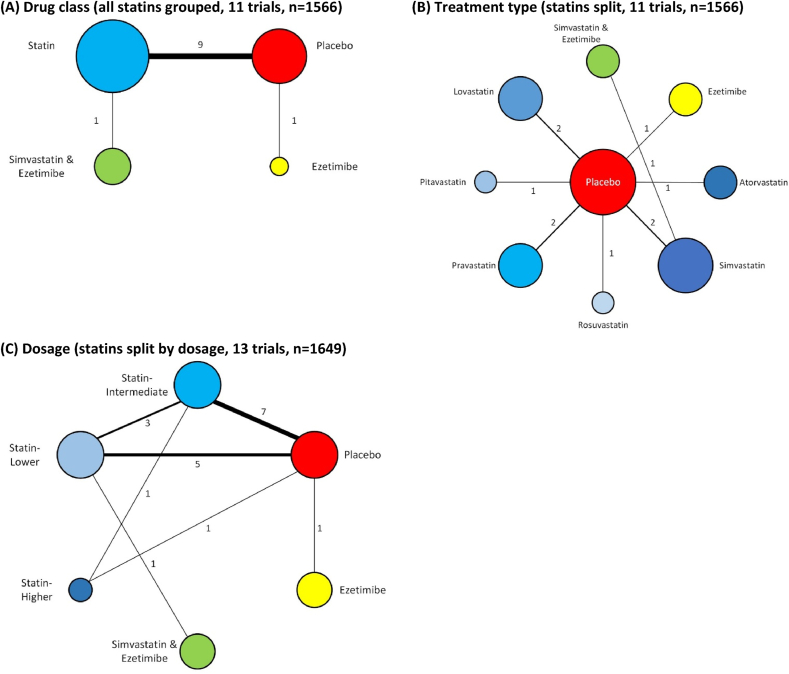
Network diagrams. Numbers indicate the number of trials informing each comparison. Larger circles indicate greater sample size.

Trials of statins, ezetimibe and statins-ezetimibe therapy were combined in three networks, which are summarised in Fig. 2.

##### 3.3.1.3 NMA results by drug class

**Fig. 3:**
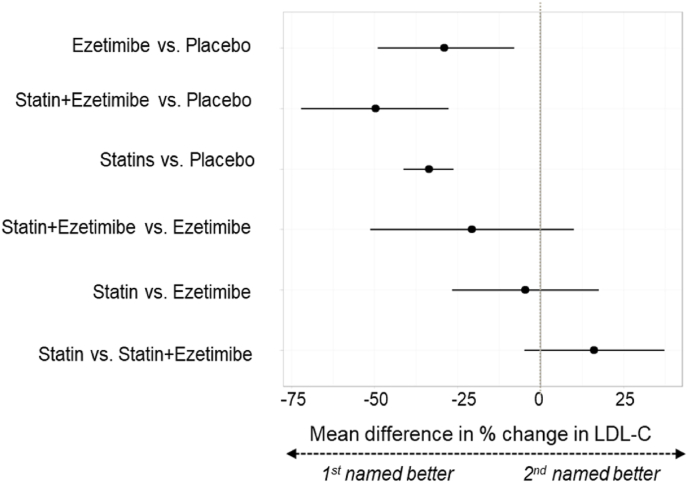
Forest plot, NMA by drug class, % LDL-C change from baseline to end of follow-up. Results expressed as mean difference in % change from baseline in LDL-C. Dots to the left of the vertical line of no effect favour the first intervention in the comparison, dots to the right favour the second. Lines represent 95 % credible intervals.

**Fig. 4:**
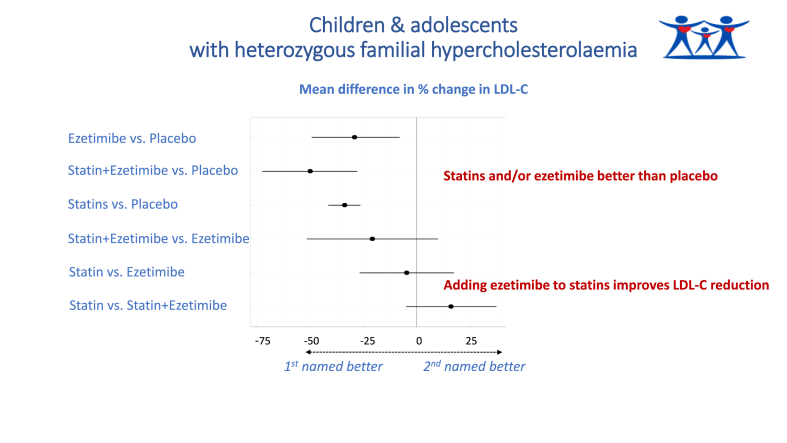
Graphical abstract.

Fig. 3 summarises the main results of the NMA for percentage reduction in LDL-C where all statins were considered equivalent in effectiveness. All effect estimates are presented in Supplementary Table 5. Statins, ezetimibe and ezetimibe combined with simvastatin were all more effective than placebo at reducing LDL-C, with effect estimates similar in magnitude to pairwise comparisons. Indirect comparisons between active treatments suggested that combination ezetimibe-simvastatin therapy may be superior to either statins alone or ezetimibe alone, but results are based on one trial. Nearly all comparisons had wide credible intervals. Fig. 4 presents an annotated graphical summary.

Supplementary Table 6 and Supplementary Figure 7 show ranking probabilities for each drug class. Statins-ezetimibe combination therapy had the highest probability of being the most effective treatment; statins monotherapy (all doses combined) was second, ezetimibe third, and placebo last.

##### 3.3.1.4 NMA results by treatment

Tables 7 and 8 and Supplementary Figures 8 and 9 present NMA results by treatment (with statins split by type) for percentage and absolute change in LDL-C (11 trials, n = 1566). All treatments were significantly superior to placebo. None of the indirect comparisons between active treatments showed any clear evidence of difference between treatments.

##### 3.3.1.5 NMA results by dosage

**Fig. 5:**
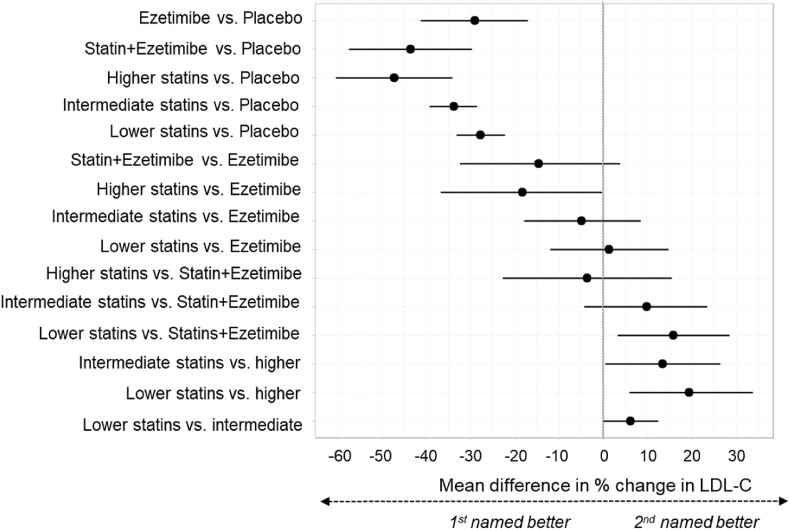
Forest plot, NMA with statin split by dosage, % LDL-C change from baseline to end of follow-up. Results expressed as mean difference in % change from baseline in LDL-C. Dots to the left of the vertical line of no effect favour the first intervention in the comparison, dots to the right favour the second. Lines represent 95 % credible intervals. “higher”, “intermediate” and “lower” refer to statin dosage.

Fig. 5, Supplementary Tables 9 and 10 present NMA results accounting for statin dosage (13 trials, n = 1649). Higher-dose statins were superior to ezetimibe, intermediate and lower-dose statins, but comparable with an intermediate-dose-statins combined with ezetimibe. There was no evidence of a difference between ezetimibe and lower or intermediate-dose statins. Intermediate dose-statins combined with ezetimibe were superior to lower-dose and probably to intermediate-dose statins alone. Intermediate-dose statins were probably superior to lower-dose statins, although the relative magnitude in percentage LDL-C reduction difference was relatively small (approximately 4.77 %).

#### 3.3.2 TC, HDL-C, TG

Pairwise meta-analyses showed that statins reduced TC 26.63 % more than placebo (95 % CI 21.48 to 31.78, I^2^ = 90 %, 8 trials). Statins increased HDL-C by 3.54 % (95 % CI 1.00 to 6.08, I^2^ = 0 %, 7 trials) decreased triglyceride by 7.97 % (95 % CI 3.07 to 12.87, I^2^ = 23 %, 7 trials), see Supplementary Figs. 10–12. Significant decreases in TC, and decreases in TG were also reported in one ezetimibe monotherapy trial and one statins-ezetimibe combination trial (see Supplementary Table 3).

#### 3.3.3 Other effectiveness results

One trial showed that pravastatin therapy led to a small and significant reduction in cIMT compared to placebo at 2 years (MD -0.01 mm; 95 % CI -0.03 to −0.00) [3] One trial showed an increase in relative flow-mediated dilatation of the brachial artery of 2.70 % (95 % CI 0.42 to 4.98) with simvastatin versus placebo at 28 weeks [34]. No studies reported quality of life outcomes.

### 3.4 Safety and maturation outcomes

There was no evidence of a difference in maturation (Tanner stage increase by one or more points) between statins and placebo (RR 0.91; 95 % CI 0.75 to 1.11, I^2^ = 0 %, 3 trials). There was no difference in AE (all events) between statins and placebo (RR 1.00, 95 % CI 0.87 to 1.16, I^2^ = 0 %, 5 trials), and no evidence of a difference in discontinuation rates (RR 0.91; 95 % CI 0.24 to 3.42, I^2^ = 0 %, 5 trials). There were no reports of rhabdomyolysis, and no evidence of a difference in incidence of myopathy (3/243 for statins *vs.* 1/207 for placebo), liver dysfunction (4/560 *vs.* 2/380) and myalgia (RR 1.99 95 % CI 0.62 to 6.43, I^2^ = 0 %, 3 trials) between statins and placebo. There was no evidence that different statins doses affected the incidence of AEs overall. There were no significant concerns about the safety and tolerability of ezetimibe monotherapy and statins + ezetimibe combination therapy, although evidence for these therapies is limited. See Supplementary Table 11 and Supplementary Figs. 13–18.

## 4 Discussion

This systematic review included 13 randomised trials of statins, ezetimibe monotherapy and statins-ezetimibe combined therapy in children with HeFH. Our analyses showed a clinically significant reduction in serum total cholesterol with LLT compared with placebo; LLTs led to increases in serum HDL cholesterol and decreases in serum triglyceride of a magnitude in line with previous reviews [[13], [14], [15]]. Evidence indicated an improvement in endothelial function with simvastatin, although this is based on one small, single trial and requires further research. LLTs were generally well-tolerated and there was no evidence that they were less safe than placebo. There was no evidence that higher-dose statins are associated with higher incidence of AEs, although this is based on a subgroup analysis of a relatively limited number of trials.

Our analyses found that all LLTs led to significant reductions in LDL-C compared with placebo. There was no conclusive evidence that one drug class or one statin type was significantly superior to another at reducing LDL-C, although statins-ezetimibe combination showed larger reductions compared with monotherapies, and there was evidence of heterogeneity due to variation in statins dosage. NMAs accounting for the effect of LLT dosage on LDL-C reductions found that higher-dose statins and intermediate-dose statins combined with ezetimibe are probably superior to intermediate-dose statins, lower-dose statins and ezetimibe. There is no evidence of a difference between higher-dose statins and intermediate-dose statins combined with ezetimibe, and any differences between the two are probably small. There is no evidence of a significant difference in efficacy between ezetimibe monotherapy and lower-to-intermediate dose statins, and any differences between these are probably small. Intermediate-dose statins may be slightly more effective than lower-dose statins, although differences are probably small.

Evidence from individual studies showed that the percentage of participants achieving the consensus LDL-C target of <2.8 mmol/L (110 mg/dL) was limited even with higher-dose statins (41 % with rosuvastatin 20 mg) [30] and with the addition of ezetimibe (63 % with simvastatin 40 mg + ezetimibe 10 mg) [17], reflecting the difficulty in achieving this target. A less strict treatment goal of <3.4 mmol/L (<130 mg/dL) was achieved by between 53 % (simvastatin 40 mg + placebo) and 77 % (with simvastatin 40 mg + ezetimibe 10 mg) [17,38].

Most children with HeFH will not receive higher-dose LLTs in practice, despite evidence indicating favourable tolerability and safety profile in the short term, the difficulty in achieving consensus LLT targets, and the increased lifetime cardiovascular risk associated with higher LDL-C from childhood [2,6,9,40,41].

### 4.1 Added value to existing literature, strengths and limitations

This is the first network meta-analysis that includes all published trials of statins and ezetimibe in children with HeFH. By broadening our scope and using advanced meta-analytic techniques, we were able to pool the totality of the RCT evidence on the main recommended LLT pharmacotherapies, including from trials comparing different statins doses, as well as trials evaluating ezetimibe with-and without statins. We included 13 trials and up to 1649 participants in our review and meta-analysis, compared with nine trials and 1177 participants in the latest Cochrane review on the topic [13]. Unlike previous evidence, our analyses produced relative effect estimates of all interventions compared with every other using both direct and indirect evidence, incorporating effects from trials with more than two arms accounting for double counting, and ranking all the interventions coherently [18]. It also allowed for formal comparisons of all LLTs for which no head-to-head randomised evidence is available, whilst preserving within-trial randomisation. Previous meta-analyses were limited in their ability to explore sources of heterogeneity [[13], [14], [15]]. In this review, NMA techniques allowed for formal comparisons of all eligible LLTs by drug class, and accounting for statin type and LLT dosage in a more reliable and accurate way to inform decision making [42]. Characteristics of participants, and the magnitude of observed LDL-C reductions were comparable with previous meta-analyses [[13], [14], [15]] and observational evidence [40,43,44]. This meta-analysis showed broadly comparable levels of LDL-C reductions and variations across LLT types and doses compared with adults [24,45].

There were no serious concerns about risk of bias, although trial reporting limited the extent to which quality could be assessed. There was no evidence of publication bias, although tests were limited by the number of studies. The NMA is limited by the number of trials, with most branches in the network informed by one or two studies, with few loops (where evidence is informed by both direct and indirect evidence). Although pooled estimates from the NMA were broadly similar to pairwise evidence, they tended to be less precise, and the limited number of loops prevented formal consistency checks. Due to limited evidence, most ranking probabilities were imprecise, and outcomes other than LDL-C could not be included in the NMA. Other potential sources of heterogeneity, such as the impact of genetic mutations on treatment response, or treatment adherence (a known concern in paediatric FH) [46], could not be explored. In the absence of individual patient data, we could not explore the effectiveness and safety of LLTs at different ages and LDL-C levels at treatment initiation.

Due to insufficient evidence, no meta-analyses were feasible for LDL-C target, cIMT, and endothelial function. Further trials are required to inform these outcomes. Although we found no evidence to suggest significant safety concerns for any of the LLTs, the evidence was insufficiently powered to detect differences in individual outcomes. The follow-up duration of the trials (maximum 2 years) was insufficient to evaluate the impact of LLTs on longer-term efficacy (including cardiovascular disease and mortality) and safety. Longer-term (20-year) observational evidence indicates that statins are both safe and effective at preventing cardiovascular events over the longer-term, and that greater reductions in LDL-C are associated with a significantly reduced risk of coronary heart disease [6,40]. There was insufficient data to explore the relative safety and benefit of LLTs in different age groups (notably children under 10 years), and only one trial had a mean age below 10.

Newer generation PCSK9i studies, inclisiran and bempedoic acid were excluded from this review, as they are not commonly used in practice. Although evolocumab and alirocumab are now licensed in children, they are not recommended for in current paediatric guidelines. Trials evaluating these therapies in paediatric FH were published in 2020 [47] and February 2024 [48]. This trial evidence indicates that evolocumab and alirocumab may significantly reduce LDL-C in HeFH paediatric patients inadequately controlled with statins [47,48], and may become a relevant adjunct to paediatric HeFH management in a subset of patients [9].

### 4.2 Clinical implications

Most trials started LLT at low doses, which is reflective of current practice. This review supports existing guidelines on paediatric HeFH management [2,7,9] and provides further evidence to support statin dose-escalation and/or addition of ezetimibe where LDL-C targets are not met, where safe, tolerated and age-appropriate.

### 4.3 Research recommendations

Future evidence is required to establish the longer-term efficacy and safety of LLTs. Direct evidence could confirm the findings from our indirect comparisons, although we recognise it is highly unlikely that direct randomised comparisons may be published in the future for all possible comparisons included in this NMA. Future research will help to inform guidance on an optimal age and LDL-C levels for treatment initiation in children and LDL-C targets [19]. Future studies should investigate the impact of dose-titration on LDL-C targets and adherence.

### 4.4 Conclusions

Statins, ezetimibe and statins-ezetimibe combination therapy all lead to clinically significant reductions in LDL-C and TC, reductions in TG, and increases in HDL-C. All LLTs were generally well-tolerated and there was no evidence that LLTs had an adverse impact on maturation or safety, although trial duration was limited. The effectiveness and safety evidence for ezetimibe is limited by the number of trials evaluating this therapy.

The magnitude of LDL-C reductions varies and may depend on dosage and the addition of ezetimibe. Higher-dose and intermediate dose statins combined with ezetimibe are probably similarly effective and superior to intermediate-and lower-dose therapies. There is no evidence of a significant difference in efficacy between ezetimibe monotherapy and lower-to intermediate-dose statins. Intermediate-dose statins may be slightly more effective than lower-dose statins, although the difference is likely to be small.

## Financial support

This project was funded by the National Institute for Health and Care Research [NIHR134993]. The views expressed are those of the authors and not necessarily those of the NIHR or the Department of Health and Social Care. SEH is an Emeritus Professor and acknowledges funding from the British Heart Foundation (BHF) (RG08/008), and the National Institute for Health Research and University College London Hospitals Biomedical Research Centre.

## CRediT authorship contribution statement

**Alexis Llewellyn:** Conceptualization, and protocol, Funding acquisition, Systematic review, Formal analysis, Writing - Original Draft, Methodology, Writing - Review & Editing. **Mark Simmonds:** Conceptualization, and protocol, Funding acquisition, Systematic review, Data curation, Formal analysis, Writing - Review & Editing. **David Marshall:** Conceptualization, and protocol, Systematic review, Writing - Review & Editing. **Melissa Harden:** Conceptualization, and protocol, Funding acquisition, Search strategies, Writing - Review & Editing. **Beth Woods:** Conceptualization, and protocol, Funding acquisition, Writing - Review & Editing. **Steve E. Humphries:** Conceptualization, and protocol, Funding acquisition, Writing - Review & Editing. **Uma Ramaswami:** Conceptualization, and protocol, Funding acquisition, Writing - Review & Editing. **Lorraine Priestley-Barnham:** Conceptualization, and protocol, Funding acquisition, Writing - Review & Editing. **Mark Fisher:** Conceptualization, and protocol, Funding acquisition, Writing - Review & Editing. **Laila J. Tata:** Conceptualization, and protocol, Funding acquisition, Writing - Review & Editing. **Nadeem Qureshi:** Conceptualization, and protocol, Funding acquisition, Writing - Review & Editing.

## Declaration of competing interest

SEH is the Medical Director of a UCL spin-off company (StoreGene) that offers genetic testing for cardiovascular risk including for FH. SEH also reports payment for expert testimony from Verve Therapeutics. BW sits on the Board of Directors for the York Health Economics Consortium (unpaid role). All other co-authors have no interests to declare.
