# Supplementary material for: Efficacy and safety of statins, ezetimibe and statins-ezetimibe therapies for children and adolescents with heterozygous familial hypercholesterolaemia: Systematic review, pairwise and network meta-analyses of randomised controlled trials
Source: Atherosclerosis. 2025 Feb;401:None. doi: 10.1016/j.atherosclerosis.2024.118598 (PMC11811749; doi:10.1016/j.atherosclerosis.2024.118598)
Supplement: Multimedia component 1 [file mmc1.docx]

# Supplementary file 1

## Literature search

The aim of the search was to systematically identify published and unpublished studies of statins or ezetimibe in children aged 0-18 years with heterozygous familial hypercholesterolaemia. An information specialist designed the search strategy in Ovid MEDLINE in consultation with the review team.

The strategy was structured using the following four concepts:

1. children (0-18 years)

AND

2. heterozygous familial hypercholestrolaemia

AND

3. statins

OR

4. ezetimibe

Search terms for each concept were identified through examination of terms used in key papers, online drug information resources, use of database thesauri and through discussion with the review team. Relevant subject headings and textword searches of the title and abstracts of records were utilised in the strategy. Retrieval was not restricted by language, date or study design.

The performance of the MEDLINE strategy was tested to ensure it retrieved all of the previous studies included in the Cochrane review by Vurio et. al.^[[1]](#footnote-1)^ The strategy was peer reviewed by a second information specialist using the PRESS checklist.^[[2]](#footnote-2)^ The final MEDLINE strategy was adapted for use in all resources searched.

The following databases were searched in December 2022:

- MEDLINE ALL (Ovid)
- Cochrane Controlled Register of Trials (Wiley)
- Cochrane Database of Systematic Reviews (Wiley)
- Embase (Ovid)
- International Health Technology Assessment (INAHTA) database
- Science Citation Index (Web of Science)

Further ongoing and unpublished studies were identified through searches of:

- ClinicalTrials.gov
- Conference Proceedings Citation Index: Science (Web of Science)
- EU Clinical Trials Register
- PROSPERO
- WHO International Clinical Trials Registry Platform portal

A search for relevant guidelines was carried out via the following websites: National Institute for Health and Clinical Excellence (NICE), Guidelines International Network (GIN) and the Trip database. Supplementary searching of the reference lists of relevant reviews and included studies was undertaken to identify any additional relevant studies.

All search results were imported into EndNote 20 reference management software (Clarivate, Philadelphia, PA) and deduplicated.

An update search of MEDLINE was carried out in January 2024 to identify recently published studies.

## Search strategies

***Database search strategies***

**MEDLINE ALL**

(includes: Epub Ahead of Print, In-Process & Other Non-Indexed Citations, Ovid MEDLINE Daily and Ovid MEDLINE)

via Ovid <http://ovidsp.ovid.com/>

Date range: 1946 to December 14, 2022

Date searched: 15^th^ December 2022

Records retrieved: 1392

An update search of MEDLINE ALL was carried out on 22^nd^ January 2024 retrieving 1444 records.

1 Hyperlipoproteinemia Type II/ (7510)

2 hyperlipidemias/ (28334)

3 hypercholesterolemia/ (27009)

4 (hyperlipoprotein?emi$ or (hyper adj3 lipoprotein?emi$) or hyperbetalipoprotein?emi$).ti,ab. (4727)

5 (hypercholesterol?emi$ or hyper cholesterol?emi$).ti,ab. (37928)

6 (hyperlipid?emi$ or hyper lipid?emi$).ti,ab. (35177)

7 heFH.ti,ab. (262)

8 heterozygous FH.ti,ab. (519)

9 1 or 2 or 3 or 4 or 5 or 6 or 7 or 8 (100191)

10 exp Child/ (2115518)

11 Adolescent/ (2196502)

12 exp Infant/ (1235768)

13 (child or children or childhood$ or infant$ or infancy or pediatric$ or paediatric$ or preschool$ or pre school$ or schoolchild$ or school age$ or schoolage$ or schoolboy$ or schoolgirl$).ti,ab. (2040862)

14 (girl or girls or boy or boys or kid or kids).ti,ab. (266629)

15 (adolesc$ or young people or young person$ or teen$ or youth$ or preteen$ or pubert$ or prepubert$ or pubescen$ or prepubescen$ or juvenil$).ti,ab. (558227)

16 (neonat$ or neo nat$ or newborn$ or new born$ or newly born$ or baby or babies).ti,ab. (484308)

17 or/10-16 (4728040)

18 9 and 17 (11494)

19 exp Hydroxymethylglutaryl-CoA Reductase Inhibitors/ (45778)

20 Atorvastatin/ (7282)

21 Fluvastatin/ (1455)

22 Lovastatin/ (4713)

23 Pravastatin/ (3527)

24 Rosuvastatin Calcium/ (2867)

25 exp Simvastatin/ (8344)

26 (HMG-CoA or hydroxymethylglutaryl CoA reductase inhibitor$ or hydroxymethylglutaryl coenzyme a inhibitor$).af. (38954)

27 (atorvastatin$ or lipitor$ or lypqozet$ or caduet$).af. (11036)

28 (fluvastatin$ or lescol$ or nandovar$ or dorisin$ or fluindostatin$).af. (2224)

29 (lovastatin$ or mevacor$ or altoprev$ or mevinolin$).af. (6334)

30 (pitavastatin$ or livalo$ or zypitamag$).af. (1147)

31 (pravastatin$ or pravachol$).af. (5095)

32 (rosuvastatin$ or crestor$ or ezallor$).af. (4462)

33 (simvastatin$ or zocor$ or flolipid$ or vytorin$ or inegy$).af. (11966)

34 statin$.af. (51062)

35 or/19-34 (78196)

36 18 and 35 (1159)

37 exp Ezetimibe/ (2503)

38 (ezetimibe$ or ezetrol$ or zetia$).af. (4477)

39 37 or 38 (4477)

40 18 and 39 (228)

41 ((lipid-modif$ or lipid-lower$) adj2 (therap$ or treatment$ or intervention$ or medication$ or drug$ or agent$)).ti,ab. (12533)

42 18 and 41 (422)

43 36 or 40 (1195)

44 42 or 43 (1402)

45 exp animals/ not humans/ (5074173)

46 44 not 45 (1392)

**Key:**

/ = subject heading (MeSH heading)

exp = exploded subject heading (MeSH heading)

$ = truncation

? = optional wild card character, stands for zero or one character within a word

ti,ab = terms in title or abstract fields

af = terms in all fields

adj3 = terms within three words of each other (any order)

**Cochrane Controlled Register of Trials (CENTRAL)**

via Wiley <http://onlinelibrary.wiley.com/>

Issue: 11 of 12, November 2022

Date searched: 15^th^ December 2022

Records retrieved: 353

#1 MeSH descriptor: [Hyperlipoproteinemia Type II] this term only 540

#2 MeSH descriptor: [Hypercholesterolemia] this term only 3617

#3 MeSH descriptor: [Hyperlipidemias] this term only 2065

#4 (hyperlipoprotein*mi* or (hyper near/3 lipoprotein*emi*) or hyperbetalipoprotein*mi*):ti,ab,kw 1498

#5 (hypercholesterol*mi* or hyper cholesterol*mi*):ti,ab,kw 8585

#6 (hyperlipid*mi* or hyper lipid*mi*):ti,ab,kw 6846

#7 heFH:ti,ab,kw 161

#8 heterozygous FH:ti,ab,kw 252

#9 #1 or #2 or #3 or #4 or #5 or #6 or #7 or #8 14929

#10 MeSH descriptor: [Child] explode all trees 62328

#11 MeSH descriptor: [Adolescent] this term only 110852

#12 MeSH descriptor: [Infant] explode all trees 35345

#13 (child or children or childhood* or infant* or infancy or pediatric* or paediatric* or preschool* or (pre next school*) or schoolchild* or (school next age*) or schoolage* or schoolboy* or schoolgirl*):ti,ab,kw 215441

#14 (girl or girls or boy or boys or kid or kids):ti,ab,kw 13045

#15 (adolesc* or (young next people) or (young next person*) or teen* or youth* or preteen* or pubert* or prepubert* or pubescen* or prepubescen* or juvenil*):ti,ab,kw 158744

#16 (neonat* or neo-nat* or newborn* or new-born* or (newly next born*) or baby or babies):ti,ab,kw 47726

#17 #10 or #11 or #12 or #13 or #14 or #15 or #16 329829

#18 #9 and #17 1083

#19 MeSH descriptor: [Hydroxymethylglutaryl-CoA Reductase Inhibitors] explode all trees 3746

#20 MeSH descriptor: [Atorvastatin] this term only 1859

#21 MeSH descriptor: [Fluvastatin] this term only 331

#22 MeSH descriptor: [Lovastatin] this term only 597

#23 MeSH descriptor: [Pravastatin] this term only 1024

#24 MeSH descriptor: [Rosuvastatin Calcium] this term only 1184

#25 MeSH descriptor: [Simvastatin] explode all trees 1868

#26 ((atorvastatin* or lipitor* or lypqozet* or caduet*)) 5860

#27 ((fluvastatin* or lescol* or nandovar* or dorisin* or fluindostatin*)) 819

#28 ((lovastatin* or mevacor* or altoprev* or mevinolin*)) 1057

#29 ((pitavastatin* or livalo* or zypitamag*)) 574

#30 ((pravastatin* or pravachol*)) 2051

#31 ((rosuvastatin* or crestor* or ezallor*)) 2783

#32 ((simvastatin* or zocor* or flolipid* or vytorin* or inegy*)) 4102

#33 ((HMG-CoA or ("hydroxymethylglutaryl CoA reductase" next inhibitor*) or ("hydroxymethylglutaryl coenzyme a" next inhibitor*))) 85

#34 (statin*) 11800

#35 #19 or #20 or #21 or #22 or #23 or #24 or #25 #26 or #27 or #28 or #29 or #30 or #31 or #32 or #33 or #34 18111

#36 #18 and #35 315

#37 MeSH descriptor: [Ezetimibe] explode all trees 821

#38 ((ezetimibe* or ezetrol* or zetia*)) 1880

#39 #37 or #38 1880

#40 #18 and #39 77

#41 ((lipid-modif* or lipid-lower*) near/2 (therap* or treatment* or intervention* or medication* or drug* or agent*)) 3175

#42 #18 and #41 89

#43 #36 or #40 or #42 in Cochrane Reviews, Cochrane Protocols 6

#44 #36 or #40 or #42 in Trials 353

**Key:**

MeSH descriptor = subject heading (MeSH heading)

* = truncation

ti,ab,kw = terms in title, abstract or keyword fields

near/3 = terms within three words of each other (any order)

next = terms are next to each other

**Cochrane Database of Systematic Reviews (CDSR)**

via Wiley <http://onlinelibrary.wiley.com/>

Issue: 12 of 12, December 2022

Date searched: 15^th^ December 2022

Records retrieved: 6

See above under CENTRAL for search strategy.

**Embase**

via Ovid <http://ovidsp.ovid.com/>

Date range: 1974 to 2022 December 14

Date searched: 15^th^ December 2022

Records retrieved: 2046

1 familial hypercholesterolemia/ (11545)

2 hypercholesterolemia/ (69241)

3 hyperlipidemia/ (85905)

4 (hyperlipoprotein?emi$ or (hyper adj3 lipoprotein?emi$) or hyperbetalipoprotein?emi$).ti,ab. (5471)

5 (hypercholesterol?emi$ or hyper cholesterol?emi$).ti,ab. (53799)

6 (hyperlipid?emi$ or hyper lipid?emi$).ti,ab. (57308)

7 heFH.ti,ab. (550)

8 heterozygous FH.ti,ab. (787)

9 or/1-8 (185499)

10 exp child/ (2972408)

11 exp adolescence/ (89310)

12 exp infant/ (1099206)

13 juvenile/ (52431)

14 (child or children or childhood$ or infant$ or infancy or pediatric$ or paediatric$ or preschool$ or pre school$ or schoolchild$ or school age$ or schoolage$ or schoolboy$ or schoolgirl$).ti,ab. (2562152)

15 (girl or girls or boy or boys or kid or kids).ti,ab. (354724)

16 (adolesc$ or young people or young person$ or teen$ or youth$ or preteen$ or pubert$ or prepubert$ or pubescen$ or prepubescen$ or juvenil$).ti,ab. (714411)

17 (neonat$ or neo nat$ or newborn$ or new born$ or newly born$ or baby or babies).ti,ab. (608785)

18 or/10-17 (4236320)

19 9 and 18 (12606)

20 exp hydroxymethylglutaryl coenzyme A reductase inhibitor/ (182223)

21 atorvastatin/ (43512)

22 atorvastatin plus ezetimibe/ (122)

23 amlodipine plus atorvastatin/ (293)

24 fluindostatin/ (10153)

25 mevinolin/ (16763)

26 pravastatin/ (21024)

27 rosuvastatin/ (18065)

28 simvastatin/ (41184)

29 ezetimibe plus simvastatin/ (1442)

30 (HMG-CoA or hydroxymethylglutaryl CoA reductase inhibitor$ or hydroxymethylglutaryl coenzyme a inhibitor$).af. (13512)

31 (atorvastatin$ or lipitor$ or lypqozet$ or caduet$).af. (44584)

32 (fluvastatin$ or lescol$ or nandovar$ or dorisin$ or fluindostatin$).af. (10334)

33 (lovastatin$ or mevacor$ or altoprev$ or mevinolin$).af. (17454)

34 (pitavastatin$ or livalo$ or zypitamag$).af. (3987)

35 (pravastatin$ or pravachol$).af. (21516)

36 (rosuvastatin$ or crestor$ or ezallor$).af. (18497)

37 (simvastatin$ or zocor$ or flolipid$ or vytorin$ or inegy$).af. (42882)

38 statin$.af. (88888)

39 or/20-38 (210417)

40 19 and 39 (1878)

41 ezetimibe/ (12247)

42 (ezetimibe$ or ezetrol$ or zetia$).af. (14069)

43 41 or 42 (14069)

44 19 and 43 (518)

45 ((lipid-modif$ or lipid-lower$) adj2 (therap$ or treatment$ or intervention$ or medication$ or drug$ or agent$)).ti,ab. (19329)

46 19 and 45 (389)

47 40 or 44 (1919)

48 46 or 47 (2068)

49 animal experiment/ (2908024)

50 human experiment/ (606206)

51 human/ (24296241)

52 50 or 51 (24302646)

53 49 not 52 (2473961)

54 (rat or rats or mouse or mice or swine or porcine or murine or sheep or lambs or pigs or piglets or rabbit or rabbits or cat or cats or dog or dogs or cattle or bovine or monkey or monkeys or trout or marmoset*).ti,ot. (2277368)

55 49 and 54 (1178440)

56 53 or 55 (2537102)

57 48 not 56 (2046)

**Key:**

/ = subject heading (Emtree heading)

exp = exploded subject heading (Emtree heading)

$ = truncation

? = optional wild card character, stands for zero or one character within a word

ti,ab = terms in title or abstract fields

af = terms in all fields

ot = terms in the other title field

adj3 = terms within three words of each other (any order)

**International Health Technology Assessment (INAHTA) database**

via <https://database.inahta.org/>

Date searched: 16^th^ December 2022

Records retrieved: 8

1. (((ezetimibe* or ezetrol* or zetia*)[Title] OR (ezetimibe* or ezetrol* or zetia*)[abs] OR (ezetimibe* or ezetrol* or zetia*)[Keywords]) OR ("Ezetimibe"[mhe]) OR ((statin*)[Title] OR (statin*)[abs] OR (statin*)[Keywords]) OR ((rosuvastatin* OR crestor* OR ezallor*)[Title] OR (rosuvastatin* OR crestor* OR ezallor*)[abs] OR (rosuvastatin* OR crestor* OR ezallor*)[Keywords]) OR ((pravastatin* OR pravachol*)[Title] OR (pravastatin* OR pravachol*)[abs] OR (pravastatin* OR pravachol*)[Keywords]) OR ((pitavastatin* OR livalo* OR zypitamag*)[Title] OR (pitavastatin* OR livalo* OR zypitamag*)[abs] OR (pitavastatin* OR livalo* OR zypitamag*)[Keywords]) OR ((lovastatin* OR mevacor* OR altoprev* OR mevinolin*)[Title] OR (lovastatin* OR mevacor* OR altoprev* OR mevinolin*)[abs] OR (lovastatin* OR mevacor* OR altoprev* OR mevinolin*)[Keywords]) OR ((fluvastatin* OR lescol* OR nandovar* OR dorisin* OR fluindostatin*)[Title] OR (fluvastatin* OR lescol* OR nandovar* OR dorisin* OR fluindostatin*)[abs] OR (fluvastatin* OR lescol* OR nandovar* OR dorisin* OR fluindostatin*)[Keywords]) OR ((atorvastatin* OR lipitor* OR lypqozet* OR caduet*)[Title] OR (atorvastatin* OR lipitor* OR lypqozet* OR caduet*)[abs] OR (atorvastatin* OR lipitor* OR lypqozet* OR caduet*)[Keywords]) OR ((HMG-CoA OR "hydroxymethylglutaryl CoA reductase" OR "hydroxymethylglutaryl coenzyme a" )[Title] OR (HMG-CoA OR "hydroxymethylglutaryl CoA reductase" OR "hydroxymethylglutaryl coenzyme a" )[abs] OR (HMG-CoA OR "hydroxymethylglutaryl CoA reductase" OR "hydroxymethylglutaryl coenzyme a" )[Keywords]) OR ("Simvastatin"[mhe]) OR ("Rosuvastatin Calcium"[mh]) OR ("Pravastatin"[mh]) OR ("Lovastatin"[mh]) OR ("Fluvastatin"[mh]) OR ("Atorvastatin"[mh]) OR ("Hydroxymethylglutaryl-CoA Reductase Inhibitors"[mhe])) AND (((neonat* OR neo-nat* OR newborn* OR new-born* OR "newly born" OR baby OR babies)[Title] OR (neonat* OR neo-nat* OR newborn* OR new-born* OR "newly born" OR baby OR babies)[abs] OR (neonat* OR neo-nat* OR newborn* OR new-born* OR "newly born" OR baby OR babies)[Keywords]) OR ((adolesc* OR "young people" OR "young person" OR teen* OR youth* OR preteen* OR pubert* OR prepubert* OR pubescen* OR prepubescen* OR juvenil*)[Title] OR (adolesc* OR "young people" OR "young person" OR teen* OR youth* OR preteen* OR pubert* OR prepubert* OR pubescen* OR prepubescen* OR juvenil*)[abs] OR (adolesc* OR "young people" OR "young person" OR teen* OR youth* OR preteen* OR pubert* OR prepubert* OR pubescen* OR prepubescen* OR juvenil*)[Keywords]) OR ((girl OR girls OR boy OR boys OR kid OR kids)[Title] OR (girl OR girls OR boy OR boys OR kid OR kids)[abs] OR (girl OR girls OR boy OR boys OR kid OR kids)[Keywords]) OR ((child OR children OR childhood* OR infant* OR infancy OR pediatric* OR paediatric* OR preschool* OR pre-school* OR schoolchild* OR school-age* OR schoolage* OR schoolboy* OR schoolgirl*)[Title] OR (child OR children OR childhood* OR infant* OR infancy OR pediatric* OR paediatric* OR preschool* OR pre-school* OR schoolchild* OR school-age* OR schoolage* OR schoolboy* OR schoolgirl*)[abs] OR (child OR children OR childhood* OR infant* OR infancy OR pediatric* OR paediatric* OR preschool* OR pre-school* OR schoolchild* OR school-age* OR schoolage* OR schoolboy* OR schoolgirl*)[Keywords]) OR ("Infant"[mhe]) OR ("Adolescent"[mh]) OR ("Child"[mhe])) AND (((heFH OR "heterozygous FH")[Title] OR (heFH OR "heterozygous FH")[abs] OR (heFH OR "heterozygous FH")[abs]) OR ((hypercholesterolemi* OR hypercholesterolaemi* OR hyperlipidemi* OR hyperlipidaemi* )[Title] OR (hypercholesterolemi* OR hypercholesterolaemi* OR hyperlipidemi* OR hyperlipidaemi*)[abs] OR (hypercholesterolemi* OR hypercholesterolaemi* OR hyperlipidemi* OR hyperlipidaemi*)[Keywords]) OR ((hyperlipoproteinemi* OR hyperlipoproteinaemi* OR hyperbetalipoproteinemi* OR hyperbetalipoproteinaemi*)[Title] OR (hyperlipoproteinemi* OR hyperlipoproteinaemi* OR hyperbetalipoproteinemi* OR hyperbetalipoproteinaemi*)[abs] OR (hyperlipoproteinemi* OR hyperlipoproteinaemi* OR hyperbetalipoproteinemi* OR hyperbetalipoproteinaemi*)[Keywords]) OR ("Hyperlipidemias"[mh]) OR ("Hypercholesterolemia"[mh]) OR ("Hyperlipoproteinemia Type II"[mh])) 4 hits

2. ((lipid modif* OR lipid lower*)[Title] OR (lipid modif* OR lipid lower*)[abs] OR (lipid modif* OR lipid lower*)[Keywords]) AND ((((neonat* OR neo-nat* OR newborn* OR new-born* OR "newly born" OR baby OR babies)[Title] OR (neonat* OR neo-nat* OR newborn* OR new-born* OR "newly born" OR baby OR babies)[abs] OR (neonat* OR neo-nat* OR newborn* OR new-born* OR "newly born" OR baby OR babies)[Keywords]) OR ((adolesc* OR "young people" OR "young person" OR teen* OR youth* OR preteen* OR pubert* OR prepubert* OR pubescen* OR prepubescen* OR juvenil*)[Title] OR (adolesc* OR "young people" OR "young person" OR teen* OR youth* OR preteen* OR pubert* OR prepubert* OR pubescen* OR prepubescen* OR juvenil*)[abs] OR (adolesc* OR "young people" OR "young person" OR teen* OR youth* OR preteen* OR pubert* OR prepubert* OR pubescen* OR prepubescen* OR juvenil*)[Keywords]) OR ((girl OR girls OR boy OR boys OR kid OR kids)[Title] OR (girl OR girls OR boy OR boys OR kid OR kids)[abs] OR (girl OR girls OR boy OR boys OR kid OR kids)[Keywords]) OR ((child OR children OR childhood* OR infant* OR infancy OR pediatric* OR paediatric* OR preschool* OR pre-school* OR schoolchild* OR school-age* OR schoolage* OR schoolboy* OR schoolgirl*)[Title] OR (child OR children OR childhood* OR infant* OR infancy OR pediatric* OR paediatric* OR preschool* OR pre-school* OR schoolchild* OR school-age* OR schoolage* OR schoolboy* OR schoolgirl*)[abs] OR (child OR children OR childhood* OR infant* OR infancy OR pediatric* OR paediatric* OR preschool* OR pre-school* OR schoolchild* OR school-age* OR schoolage* OR schoolboy* OR schoolgirl*)[Keywords]) OR ("Infant"[mhe]) OR ("Adolescent"[mh]) OR ("Child"[mhe])) AND (((heFH OR "heterozygous FH")[Title] OR (heFH OR "heterozygous FH")[abs] OR (heFH OR "heterozygous FH")[abs]) OR ((hypercholesterolemi* OR hypercholesterolaemi* OR hyperlipidemi* OR hyperlipidaemi* )[Title] OR (hypercholesterolemi* OR hypercholesterolaemi* OR hyperlipidemi* OR hyperlipidaemi*)[abs] OR (hypercholesterolemi* OR hypercholesterolaemi* OR hyperlipidemi* OR hyperlipidaemi*)[Keywords]) OR ((hyperlipoproteinemi* OR hyperlipoproteinaemi* OR hyperbetalipoproteinemi* OR hyperbetalipoproteinaemi*)[Title] OR (hyperlipoproteinemi* OR hyperlipoproteinaemi* OR hyperbetalipoproteinemi* OR hyperbetalipoproteinaemi*)[abs] OR (hyperlipoproteinemi* OR hyperlipoproteinaemi* OR hyperbetalipoproteinemi* OR hyperbetalipoproteinaemi*)[Keywords]) OR ("Hyperlipidemias"[mh]) OR ("Hypercholesterolemia"[mh]) OR ("Hyperlipoproteinemia Type II"[mh]))) 4 hits

**Key:**

[abs] = abstract

[mh] = subject heading (MeSH heading)

[mhe] = exploded subject heading (MeSH heading)

* = truncation

**Science Citation Index**

via Web of Science, Clarivate Analytics <https://clarivate.com/>

Date range: 1900 - present

Date searched: 15^th^ December 2022

Records retrieved: 805

1: TS=(child or children or childhood* or infant* or infancy or pediatric* or paediatric* or preschool* or pre school* or schoolchild* or school age* or schoolage* or schoolboy* or schoolgirl*) 2065506

2: TS=(girl or girls or boy or boys or kid or kids) 200039

3: TS=(adolesc* or "young people" or "young person"* or teen* or youth* or preteen* or pubert* or prepubert* or pubescen* or prepubescen* or juvenil*) 652343

4: TS=(neonat* or neo-nat* or newborn* or "new born*" or "newly born*" or baby or babies) 458969

5: #1 OR #2 OR #3 OR #4 2777929

6: TS=((hyperlipoprotein$emi* or (hyper NEAR/3 lipoprotein$emi*) or hyperbetalipoprotein$emi*) ) 4596

7: TS=(hypercholesterol$emi* or hyper cholesterol$emi*) 49981

8: TS=(hyperlipid$emi* or hyper lipid$emi*) 37320

9: TS=heFH 250

10: TS=("heterozygous FH") 430

11: #6 OR #7 OR #8 OR #9 OR #10 85457

12: #5 AND #11 5172

13: TS=(statin*) 63558

14: TS=(HMG-CoA or "hydroxymethylglutaryl CoA reductase inhibitor*" or "hydroxymethylglutaryl coenzyme a inhibitor*") 12376

15: TS=(atorvastatin* or Lipitor* or lypqozet* or caduet*) 17587

16: TS=(Fluvastatin* or lescol* or nandovar* or dorisin* or fluindostatin*) 2846

17: TS=(lovastatin* or mevacor* or altoprev* or mevinolin*) 7433

18: TS=(pitavastatin* or livalo* or zypitamag*) 1464

19: TS=(pravastatin* or Pravachol*) 9156

20: TS=(rosuvastatin* or crestor* or ezallor*) 6439

21: TS=(simvastatin* or zocor* or flolipid* or vytorin* or inegy*) 17620

22: #21 OR #20 OR #19 OR #18 OR #17 OR #16 OR #15 OR #14 OR #13 92863

23: #22 AND #12 711

24: TS=(ezetimibe* or ezetrol* or zetia*) 4949

25: #24 AND #12 125

26: TS=((lipid-modif* or lipid-lower*) NEAR/2 (therap* or treatment* or intervention* or medication* or drug* or agent*)) 14056

27: #26 AND #12 234

28: #27 OR #25 OR #23 821

29: TI=(animal or animals or rat or rats or mouse or mice or rodent or rodents or porcine or murine or sheep or lamb or lambs or ewe or ewes or pig or pigs or piglet or piglets or sow or sows or minipig or minipigs or rabbit or rabbits or kitten or kittens or dog or dogs or puppy or puppies or monkey or monkeys or horse or horses or foal or foals or equine or calf or calves or cattle or heifer or heifers or hamster or hamsters or chicken or chickens or livestock) 3108081

30: #28 NOT #29 805

**Key:**

TS = topic tag; searches in title, abstract, author keywords and keywords plus fields

* = truncation

$ = represents zero or one character

NEAR/3 = terms within three words of each other (any order)

***On-going, unpublished or grey literature search strategies***

**ClinicalTrials.gov**

<https://clinicaltrials.gov/ct2/>

Date searched: 16^th^ December 2022

Records retrieved: 126

Advanced search page used with age group child (birth-17) box ticked.

1. 36 Studies found for: statin | hypercholesterolemia OR hypercholesterolaemia OR hyperlipoproteinemia OR hyperlipoproteinaemia OR hyperlipidemia OR hyperlipidaemia OR heFH OR "heterozygous FH" | Child

2. 15 Studies found for: atorvastatin OR lipitor OR lypqozet OR caduet | hypercholesterolemia OR hypercholesterolaemia OR hyperlipoproteinemia OR hyperlipoproteinaemia OR hyperlipidemia OR hyperlipidaemia OR heFH OR "heterozygous FH" | Child

3. 3 Studies found for: fluvastatin OR lescol OR nandovar OR dorisin OR fluindostatin | hypercholesterolemia OR hypercholesterolaemia OR hyperlipoproteinemia OR hyperlipoproteinaemia OR hyperlipidemia OR hyperlipidaemia OR heFH OR "heterozygous FH" | Child

4. 3 Studies found for: lovastatin OR mevacor OR altoprev OR mevinolin | hypercholesterolemia OR hypercholesterolaemia OR hyperlipoproteinemia OR hyperlipoproteinaemia OR hyperlipidemia OR hyperlipidaemia OR heFH OR "heterozygous FH" | Child

5. 1 Study found for: pitavastatin OR livalo OR zypitamag | hypercholesterolemia OR hypercholesterolaemia OR hyperlipoproteinemia OR hyperlipoproteinaemia OR hyperlipidemia OR hyperlipidaemia OR heFH OR "heterozygous FH" | Child

6. 4 Studies found for: pravastatin OR Pravachol | hypercholesterolemia OR hypercholesterolaemia OR hyperlipoproteinemia OR hyperlipoproteinaemia OR hyperlipidemia OR hyperlipidaemia OR heFH OR "heterozygous FH" | Child

7. 8 Studies found for: rosuvastatin OR crestor OR ezallor | hypercholesterolemia OR hypercholesterolaemia OR hyperlipoproteinemia OR hyperlipoproteinaemia OR hyperlipidemia OR hyperlipidaemia OR heFH OR "heterozygous FH" | Child

8. 10 Studies found for: simvastatin OR zocor OR flolipid OR vytorin OR inegy | hypercholesterolemia OR hypercholesterolaemia OR hyperlipoproteinemia OR hyperlipoproteinaemia OR hyperlipidemia OR hyperlipidaemia OR heFH OR "heterozygous FH" | Child

9. 18 Studies found for: ezetimibe OR ezetrol OR zetia | hypercholesterolemia OR hypercholesterolaemia OR hyperlipoproteinemia OR hyperlipoproteinaemia OR hyperlipidemia OR hyperlipidaemia OR heFH OR "heterozygous FH" | Child

10. 28 Studies found for: "lipid lowering" OR "lipid modifying" | hypercholesterolemia OR hypercholesterolaemia OR hyperlipoproteinemia OR hyperlipoproteinaemia OR hyperlipidemia OR hyperlipidaemia OR heFH OR "heterozygous FH" | Child

**Conference Proceedings Citation Index – Science (CPCI-Science)**

via Web of Science, Clarivate Analytics <https://clarivate.com/>

Date range: 1990 - present (CPCI-Science)

Date searched: 16^th^ December 2022

1: TS=(child or children or childhood* or infant* or infancy or pediatric* or paediatric* or preschool* or pre school* or schoolchild* or school age* or schoolage* or schoolboy* or schoolgirl*) 197040

2: TS=(girl or girls or boy or boys or kid or kids) 10811

3: TS=(adolesc* or "young people" or "young person"* or teen* or youth* or preteen* or pubert* or prepubert* or pubescen* or prepubescen* or juvenil*) 53013

4: TS=(neonat* or neo-nat* or newborn* or "new born*" or "newly born*" or baby or babies 33311

5: #4 OR #3 OR #2 OR #1262417

6: TS=((hyperlipoprotein$emi* or (hyper NEAR/3 lipoprotein$emi*) or hyperbetalipoprotein$emi*)) 183

7: TS=(hypercholesterol$emi* or "hyper cholesterol$emi*") 4604

8: TS=(hyperlipid$emi* or "hyper lipid$emi*") 2804

9: TS=heFH10

10: TS=("heterozygous FH") 13

11: #10 OR #9 OR #8 OR #7 OR #6 7210

12: #11 AND #5 366

13: TS=(statin*) 7679

14: TS=(HMG-CoA or "hydroxymethylglutaryl CoA reductase inhibitor*" or "hydroxymethylglutaryl coenzyme a inhibitor*") 893

15: TS=(atorvastatin* or Lipitor* or lypqozet* or caduet*) 2055

16: TS=(Fluvastatin* or lescol* or nandovar* or dorisin* or fluindostatin*) 346

17: TS=(lovastatin* or mevacor* or altoprev* or mevinolin*) 532

18: TS=(pitavastatin* or livalo* or zypitamag*) 235

19: TS=(pravastatin* or Pravachol*) 877

20: TS=(rosuvastatin* or crestor* or ezallor*) 774

21: TS=(simvastatin* or zocor* or flolipid* or vytorin* or inegy*) 1800

22: #21 OR #20 OR #19 OR #18 OR #17 OR #16 OR #15 OR #14 OR #13 12821

23: #12 AND #22 43

24: TS=(ezetimibe* or ezetrol* or zetia*) 553

25: #24 AND #12 3

26: TS=((lipid-modif* or lipid-lower*) NEAR/2 (therap* or treatment* or intervention* or medication* or drug* or agent*)) 1055

27: #26 AND #12 7

28: #27 OR #25 OR #23 49

**Key:**

TS = topic tag; searches in title, abstract, author keywords and keywords plus fields

* = truncation

$ = represents zero or one character

NEAR/3 = terms within three words of each other (any order)

**EU Clinical Trials Register**

<https://www.clinicaltrialsregister.eu/ctr-search/search>

Search date: 16th December 2022

Records retrieved: 67

Advanced search with following age categories selected – adolescent, children, infant and toddler, newborn, preterm newborn infants, under 18.

1. 22 result(s) found for: (hypercholesterolemia OR hypercholesterolaemia OR hyperlipoproteinemia OR hyperlipoproteinaemia OR hyperlipidemia OR hyperlipidaemia OR heFH OR "heterozygous FH") AND (statin OR statins)

2. 4 result(s) found for: (hypercholesterolemia OR hypercholesterolaemia OR hyperlipoproteinemia OR hyperlipoproteinaemia OR hyperlipidemia OR hyperlipidaemia OR heFH OR "heterozygous FH") AND (atorvastatin OR lipitor OR lypqozet OR caduet)

3. 0 results found for: (hypercholesterolemia OR hypercholesterolaemia OR hyperlipoproteinemia OR hyperlipoproteinaemia OR hyperlipidemia OR hyperlipidaemia OR heFH OR "heterozygous FH") AND (fluvastatin OR lescol OR nandovar OR dorisin OR fluindostatin)

4. 1 result(s) found for: (hypercholesterolemia OR hypercholesterolaemia OR hyperlipoproteinemia OR hyperlipoproteinaemia OR hyperlipidemia OR hyperlipidaemia OR heFH OR "heterozygous FH") AND (lovastatin OR mevacor OR altoprev OR mevinolin)

5. 2 result(s) found for: (hypercholesterolemia OR hypercholesterolaemia OR hyperlipoproteinemia OR hyperlipoproteinaemia OR hyperlipidemia OR hyperlipidaemia OR heFH OR "heterozygous FH") AND (pitavastatin OR livalo OR zypitamag)

6. 5 result(s) found for: (hypercholesterolemia OR hypercholesterolaemia OR hyperlipoproteinemia OR hyperlipoproteinaemia OR hyperlipidemia OR hyperlipidaemia OR heFH OR "heterozygous FH") AND (pravastatin OR Pravachol OR rosuvastatin OR crestor OR ezallor)

7. 7 result(s) found for: (hypercholesterolemia OR hypercholesterolaemia OR hyperlipoproteinemia OR hyperlipoproteinaemia OR hyperlipidemia OR hyperlipidaemia OR heFH OR "heterozygous FH") AND (simvastatin OR zocor OR flolipid OR vytorin OR inegy OR ezetimibe OR ezetrol OR zetia)

8. 26 result(s) found for: (hypercholesterolemia OR hypercholesterolaemia OR hyperlipoproteinemia OR hyperlipoproteinaemia OR hyperlipidemia OR hyperlipidaemia OR heFH OR "heterozygous FH") AND ("lipid lowering" OR "lipid modifying")

9. 0 results found for: (hypercholesterolemia OR hypercholesterolaemia OR hyperlipoproteinemia OR hyperlipoproteinaemia OR hyperlipidemia OR hyperlipidaemia OR heFH OR "heterozygous FH") AND (HMG-CoA OR "hydroxymethylglutaryl CoA reductase inhibitor" OR "hydroxymethylglutaryl coenzyme a inhibitor")

10. 0 results found for: (hypercholesterolemia OR hypercholesterolaemia OR hyperlipoproteinemia OR hyperlipoproteinaemia OR hyperlipidemia OR hyperlipidaemia OR heFH OR "heterozygous FH") AND (HMG-CoA OR "hydroxymethylglutaryl CoA reductase inhibitors" OR "hydroxymethylglutaryl coenzyme a inhibitors")

**PROSPERO**

via <https://www.crd.york.ac.uk/prospero/>

Date searched: 19^th^ December 2022

Records retrieved: 44

#1 MeSH DESCRIPTOR Hyperlipoproteinemia Type II 14

#2 MeSH DESCRIPTOR hyperlipidemias 49

#3 MeSH DESCRIPTOR hypercholesterolemia 24

#4 (hyperlipoprotein*emi* or (hyper adj3 lipoprotein*emi*) or hyperbetalipoprotein*emi*) 23

#5 hypercholesterol*emi* or hyper cholesterol*emi* 466

#6 hyperlipid*emi* or hyper lipid*emi* 812

#7 heFH or "heterozygous FH" 21

#8 #1 OR #2 OR #3 OR #4 OR #5 OR #6 OR #7 1192

#9 MeSH DESCRIPTOR Child EXPLODE ALL TREES 7034

#10 MeSH DESCRIPTOR Adolescent 3344

#11 MeSH DESCRIPTOR infant EXPLODE ALL TREES 1950

#12 child or children or childhood* or infant* or infancy or pediatric* or paediatric* or preschool* or pre-school* or schoolchild* or school-age* or schoolage* or schoolboy* or schoolgirl* 49068

#13 girl or girls or boy or boys or kid or kids 1864

#14 adolesc* or "young people" or "young person" or "young persons" or teen* or youth* or preteen* or pubert* or prepubert* or pubescen* or prepubescen* or juvenil* 22301

#15 neonat* or neo-nat* or newborn* or "new born" or "new borns" or "newly born" or baby or babies 8422

#16 #9 OR #10 OR #11 OR #12 OR #13 OR #14 OR #15 58311

#17 #8 AND #16 280

#18 MeSH DESCRIPTOR Hydroxymethylglutaryl-CoA Reductase Inhibitors EXPLODE ALL TREES 231

#19 MeSH DESCRIPTOR Atorvastatin 2

#20 MeSH DESCRIPTOR Fluvastatin 0

#21 MeSH DESCRIPTOR Lovastatin 0

#22 MeSH DESCRIPTOR Pravastatin 0

#23 MeSH DESCRIPTOR Rosuvastatin Calcium 4

#24 MeSH DESCRIPTOR Simvastatin EXPLODE ALL TREES 9

#25 HMG-CoA or ("hydroxymethylglutaryl CoA reductase" adj inhibitor*) or ("hydroxymethylglutaryl coenzyme a" adj inhibitor*) 131

#26 atorvastatin* or Lipitor* or lypqozet* or caduet* 188

#27 Fluvastatin* or lescol* or nandovar* or dorisin* or fluindostatin* 104

#28 lovastatin* or mevacor* or altoprev* or mevinolin* 108

#29 pitavastatin* or livalo* or zypitamag* 77

#30 pravastatin* or Pravachol* 116

#31 rosuvastatin* or crestor* or ezallor* 134

#32 simvastatin* or zocor* or flolipid* or vytorin* or inegy* 168

#33 statin* 1435

#34 #18 OR #19 OR #20 OR #21 OR #22 OR #23 OR #24 OR #25 OR #26 OR #27 OR #28 OR #29 OR #30 OR #31 OR #32 OR #33 1510

#35 #17 AND #34 35

#36 MeSH DESCRIPTOR Ezetimibe EXPLODE ALL TREES 9

#37 ezetimibe* or ezetrol* or zetia* 113

#38 #36 OR #37 114

#39 #17 AND #38 8

#40 #35 OR #39 35

#41 (lipid-modif* or lipid-lower*) adj2 (therap* or treatment* or intervention* or medication* or drug* or agent* ) 345

#42 #17 AND #41 25

#43 #42 OR #40 44

**Key:**

MeSH DESCRIPTOR = subject heading (MeSH heading)

* = truncation

adj3 = terms within 3 words of each other (order specified)

**WHO International Clinical Trials Registry Platform (ICTRP)**

<https://trialsearch.who.int/AdvSearch.aspx>

Date searched: 23^rd^ May 2022

Records retrieved: 45

Advanced search screen. Recruitment status set to ALL, restricted to clinical trials in children.

1.Condition field: (hypercholesterolemia OR hypercholesterolaemia OR hyperlipoproteinemia OR hyperlipoproteinaemia OR hyperlipidemia OR hyperlipidaemia OR heFH OR "heterozygous FH")

Intervention field: (statin)

0 records

3. Condition field: (hypercholesterolemia OR hypercholesterolaemia OR hyperlipoproteinemia OR hyperlipoproteinaemia OR hyperlipidemia OR hyperlipidaemia OR heFH OR "heterozygous FH")

Intervention field: (atorvastatin OR lipitor OR lypqozet OR caduet)

27 records for 12 trials found

4. Condition field: (hypercholesterolemia OR hypercholesterolaemia OR hyperlipoproteinemia OR hyperlipoproteinaemia OR hyperlipidemia OR hyperlipidaemia OR heFH OR "heterozygous FH")

Intervention field: (fluvastatin OR lescol OR nandovar OR dorisin OR fluindostatin

2 records for 2 trials found

5. Condition field: (hypercholesterolemia OR hypercholesterolaemia OR hyperlipoproteinemia OR hyperlipoproteinaemia OR hyperlipidemia OR hyperlipidaemia OR heFH OR "heterozygous FH")

Intervention field: lovastatin OR mevacor OR altoprev OR mevinolin

1 records for 1 trial found

6. Condition field: (hypercholesterolemia OR hypercholesterolaemia OR hyperlipoproteinemia OR hyperlipoproteinaemia OR hyperlipidemia OR hyperlipidaemia OR heFH OR "heterozygous FH")

Intervention field: (pitavastatin OR livalo OR zypitamag OR pravastatin OR Pravachol OR rosuvastatin OR crestor OR ezallor)

32 records for 11 trials found

7. Condition field: (hypercholesterolemia OR hypercholesterolaemia OR hyperlipoproteinemia OR hyperlipoproteinaemia OR hyperlipidemia OR hyperlipidaemia OR heFH OR "heterozygous FH")

Intervention field: (simvastatin OR zocor OR flolipid OR vytorin OR inegy OR ezetimibe OR ezetrol OR zetia)

39 records for 18 trials found

8. Condition field: (hypercholesterolemia OR hypercholesterolaemia OR hyperlipoproteinemia OR hyperlipoproteinaemia OR hyperlipidemia OR hyperlipidaemia OR heFH OR "heterozygous FH")

Intervention field: "lipid lowering" OR "lipid modifying"

2 records for 1 trials found

***Guideline website searches***

Simple searches were carried out via the guideline websites listed below and any results were browsed for relevance. Relevant guidelines identified were checked against the endnote library of results and added to the library if they had not already been found through previous searches.

**GIN international guideline library**

<https://guidelines.ebmportal.com/>

Date searched: 21^st^ December 2022

Records retrieved: 7

1. "familial hypercholesterolemia" – 0 hits

2. "familial hypercholesterolaemia" – 0 hits

3. hypercholesterolemia – 1 hit

4. hypercholesterolaemia – 2 hits, 1 was a duplicate

5. heterozygous FH – 0 hits

6. HeFH - 0 hits

7. Hyperlipoproteinemia – 0 hits

8. Hyperlipoproteinaemia - 0 hits

9. hyperlipidemia – 4 results, 2 were duplicates

10. hyperlipidaemia – 0 hits

11. dyslipidaemia – 3 hits, 2 were duplicates

12. dyslipidemia – 4 hits, 2 were duplicates

**National Institute of health and Care Excellence (NICE)**

<https://www.nice.org.uk/>

Date searched: 21^st^ December 2022

Records retrieved: 4

1. “Familial hypercholesterolaemia”, limited to guidance documents – 11 results browsed for relevance, 4 relevant.

**Trip database**

<https://www.tripdatabase.com/>

Date searched: 21^st^ December 2022

Records retrieved: 7

hypercholesterolaemia OR hypercholstolerolemia OR hefh OR "heterozygous fh" OR hyperlipoproteinemia OR hyperlipoproteinaemia OR hyperlipidemia OR hyperlipidaemia - 13 hits browsed for relevance - 2 not relevant – screening only, 4 links not working, 7 relevant.

# Supplementary file 2

Table 1 LLT dose classification

|  |  |  |
| --- | --- | --- |
| **Treatment** | **Dosage** | **Classification** |
| Atorvastatin | 10-20mg/day | intermediate to higher |
|  |  |  |
| Lovastatin | 10mg/day | lower |
|  | 20mg/day | lower |
|  | 30mg/day | intermediate |
|  | 40mg/day | intermediate |
|  |  |  |
| Pitavastatin | 1mg/day | lower |
|  | 2mg/day | lower |
|  | 4mg/day | intermediate |
|  |  |  |
| Pravastatin | 5mg/day | lower |
|  | 10mg/day | lower |
|  | 20mg/day | lower |
|  | 40mg/day | lower |
|  |  |  |
| Rosuvastatin | 5mg/day | intermediate |
|  | 10mg/day | higher |
|  | 20mg/day | higher |
|  |  |  |
| Simvastatin | 10mg/day | lower |
|  | 20mg/day | intermediate |
|  | 40mg/day | intermediate |
|  |  |  |
| Simvastatin +Ezetimibe | 10mg & 10mg  20mg & 10mg  40mg & 10mg | higher  higher  higher |
|  |  |  |
| Ezetimibe | 10mg | lower |

1. Vuorio A, Kuoppala J, Kovanen P, Humphries S, Tonstad S, Wiegman A, et al. Statins for children with familial hypercholesterolemia. *Cochrane Database of Systematic Reviews* 2019;Issue 11:Art. No.: CD006401 [↑](#footnote-ref-1)
2. McGowan J, Sampson M, Salzwedel DM, Cogo E, Foerster V, Lefebvre C. PRESS Peer Review of Electronic Search Strategies: 2015 guideline statement. *J Clin Epidemiol* 2016;**75**:40-6. [↑](#footnote-ref-2)
