# Supplementary material for: Efficacy and safety of statins, ezetimibe and statins-ezetimibe therapies for children and adolescents with heterozygous familial hypercholesterolaemia: Systematic review, pairwise and network meta-analyses of randomised controlled trials
Source: Atherosclerosis. 2025 Feb;401:None. doi: 10.1016/j.atherosclerosis.2024.118598 (PMC11811749; doi:10.1016/j.atherosclerosis.2024.118598)

# Supplementary figures

[Supplementary Figure 1 Study selection process (PRISMA flow diagram) 2](#_Toc169776297)

[Supplementary Figure 2 Forest plot, statins vs. placebo, absolute change from baseline in serum low-density lipoprotein cholesterol (LDL-C) at end of follow-up 2](#_Toc169776298)

[Supplementary Figure 3 Forest plot, statins vs. placebo, % change from baseline in serum LDL-C, subgroup by dose intensity 3](#_Toc169776299)

[Supplementary Figure 4 Forest plot, statins vs. placebo, % change from baseline in serum LDL-C, subgroup by trial duration 4](#_Toc169776300)

[Supplementary Figure 5 Forest plot, statins vs. placebo, % change from baseline in serum LDL-C, subgroup by statin type 5](#_Toc169776301)

[Supplementary Figure 6 Forest plot, statins vs. placebo, % change from baseline in serum LDL-C, subgroup by baseline-LDL-C 6](#_Toc169776302)

[Supplementary Figure 7 NMA ranking probabilities by drug class, %change in serum LDL-C at end of follow-up 7](#_Toc169776303)

[Figure 8 Forest plot, NMA by treatment (statins split by type), % LDL-C change at end of follow-up 8](#_Toc169776304)

[Supplementary Figure 9 Ranking probabilities by LLT and statin dose intensity, %change in serum LDL-C at end of follow-up 9](#_Toc169776305)

[Supplementary Figure 10 Forest plot, statins vs. placebo, % serum total cholesterol change from baseline at end of follow-up 9](#_Toc169776306)

[Supplementary Figure 11 Forest plot, statins vs. placebo, % serum high-density lipoprotein cholesterol change from baseline at end of follow-up 10](#_Toc169776307)

[Supplementary Figure 12 Forest plot, statins vs. placebo, % triglyceride change from baseline at end of follow-up 10](#_Toc169776308)

[Supplementary Figure 13 Forest plot, statins vs. placebo, change in Tanner stage from baseline at end of follow-up 10](#_Toc169776309)

[Supplementary Figure 14 Forest plot, statins vs. placebo, any adverse event by end of follow-up 11](#_Toc169776310)

[Supplementary Figure 15 Forest plot, statins vs. placebo, any AE by dose intensity by end of follow-up 11](#_Toc169776311)

[Supplementary Figure 16 Forest plot, statins vs. placebo, any serious adverse event by end of follow-up 12](#_Toc169776312)

[Supplementary Figure 17 Forest plot, statins vs. placebo, discontinuation due to adverse event by end of follow-up 12](#_Toc169776313)

[Supplementary Figure 18 Forest plot, statins vs. placebo, myalgia by end of follow-up 12](#_Toc169776314)

[Figure 19 Contour-enhanced funnel plot, statins vs. placebo, % change from baseline in serum LDL-C 13](#_Toc169776315)

Supplementary Figure 1 Study selection process (PRISMA flow diagram)

Supplementary Figure 2 Forest plot, statins vs. placebo, absolute change from baseline in serum low-density lipoprotein cholesterol (LDL-C) at end of follow-up


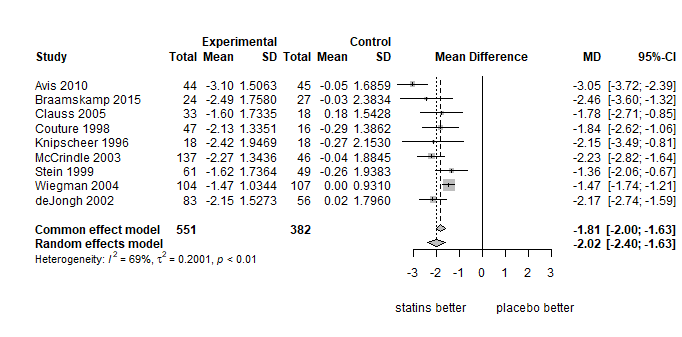


Supplementary Figure 3 Forest plot, statins vs. placebo, % change from baseline in serum LDL-C, subgroup by dose intensity


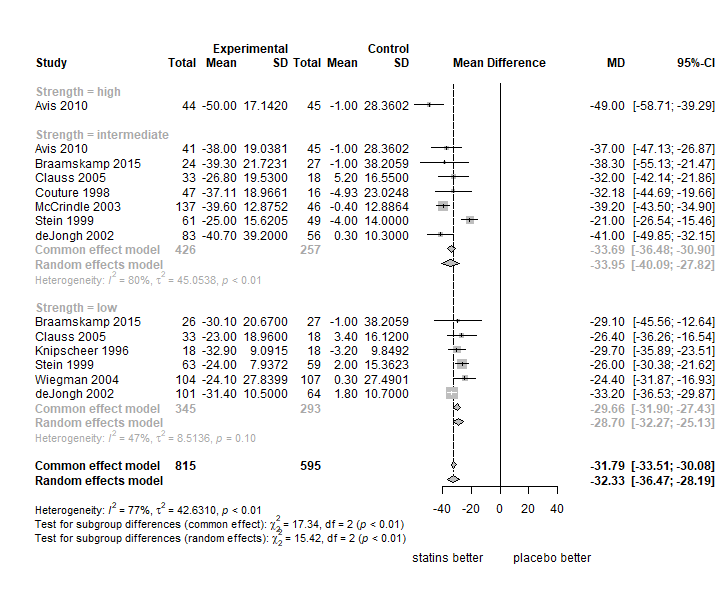


Supplementary Figure 4 Forest plot, statins vs. placebo, % change from baseline in serum LDL-C, subgroup by trial duration


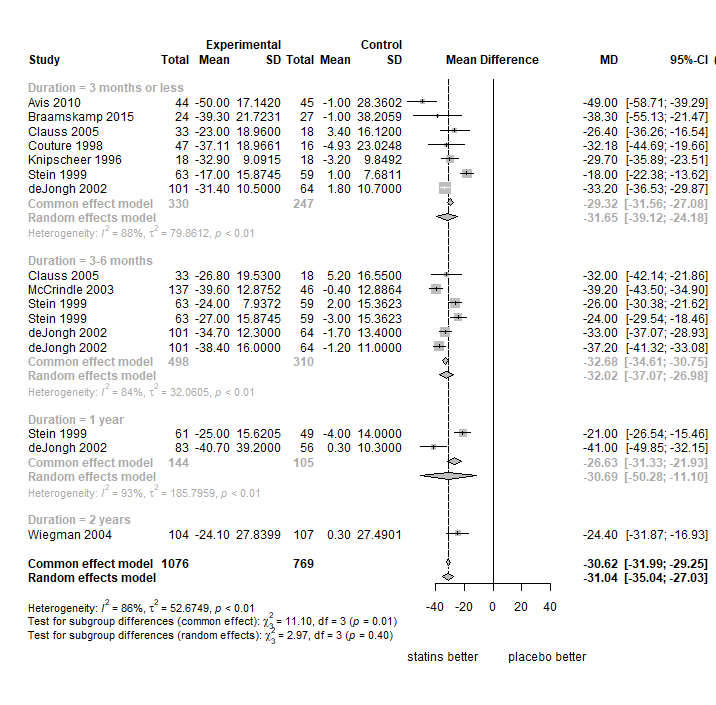


Supplementary Figure 5 Forest plot, statins vs. placebo, % change from baseline in serum LDL-C, subgroup by statin type


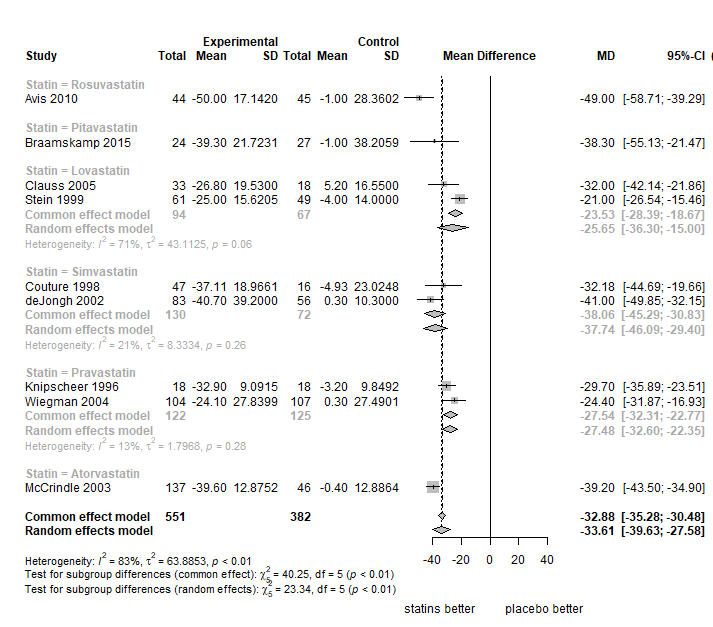


Supplementary Figure 6 Forest plot, statins vs. placebo, % change from baseline in serum LDL-C, subgroup by baseline-LDL-C


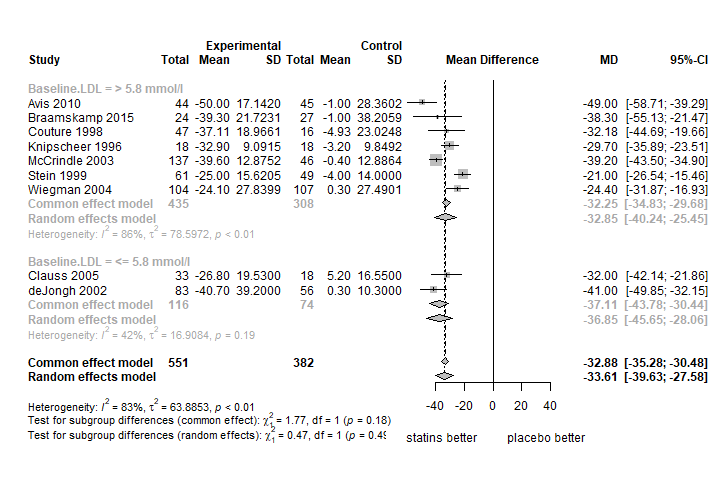


Supplementary Figure 7 NMA ranking probabilities by drug class, %change in serum LDL-C at end of follow-up


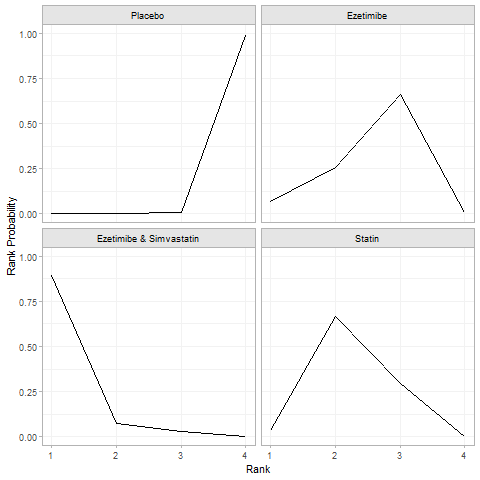


*Y axis presents the probability (ranging from 0 to 100%) of having a given rank on the X axis (ranging from 1 to 4) with 1 being the best and 4 the worst.

Figure 8 Forest plot, NMA by treatment (statins split by type), % LDL-C change at end of follow-up


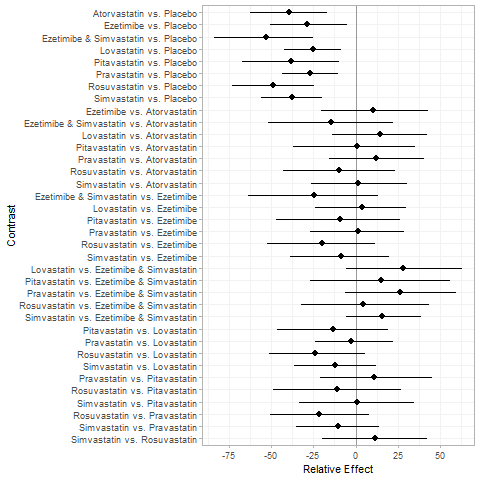


Results expressed as mean difference in % change from baseline in LDL-C. Dots to the left of the line of no effect favour the first intervention in the comparison, dots to the right favour the second. Lines represent 95% credible intervals.

Supplementary Figure 9 Ranking probabilities by LLT and statin dose intensity, %change in serum LDL-C at end of follow-up


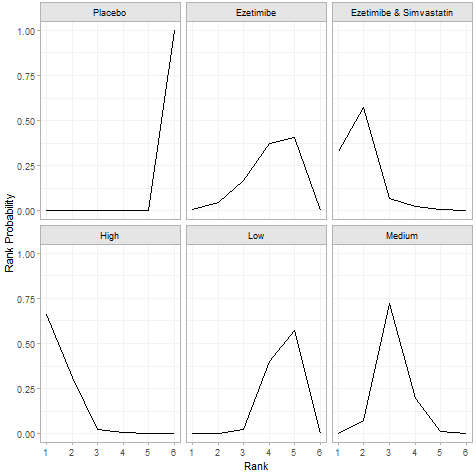


*Y axis presents the probability (ranging from 0 to 100%) of having a given rank on the X axis (ranging from 1 to 6), with 1 being the best and 6 the worst.

Supplementary Figure 10 Forest plot, statins vs. placebo, % serum total cholesterol change from baseline at end of follow-up


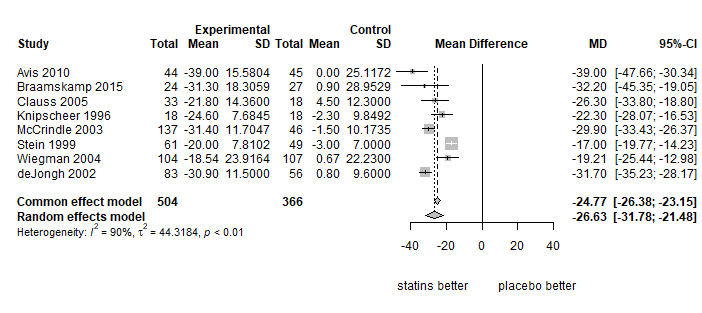


Supplementary Figure 11 Forest plot, statins vs. placebo, % serum high-density lipoprotein cholesterol change from baseline at end of follow-up


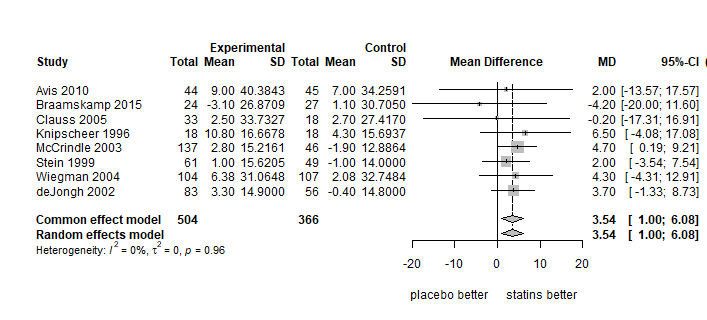


Supplementary Figure 12 Forest plot, statins vs. placebo, % triglyceride change from baseline at end of follow-up


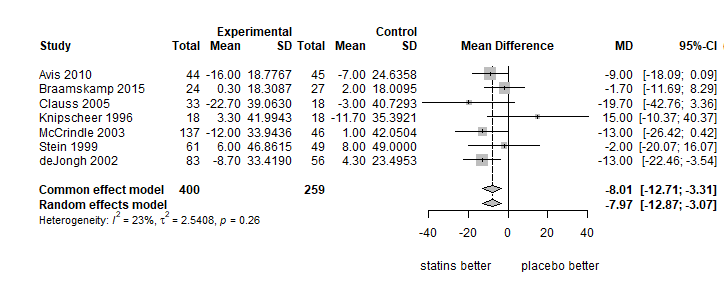


Supplementary Figure 13 Forest plot, statins vs. placebo, change in Tanner stage from baseline at end of follow-up


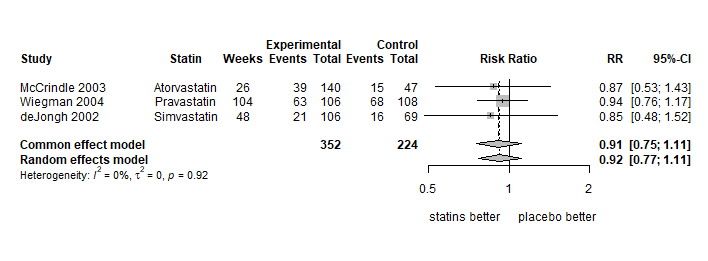


Supplementary Figure 14 Forest plot, statins vs. placebo, any adverse event by end of follow-up
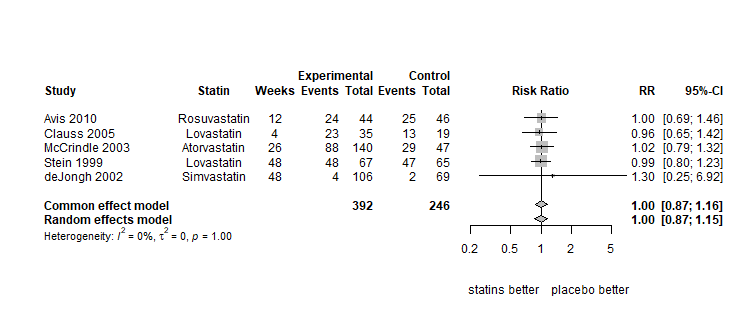


Supplementary Figure 15 Forest plot, statins vs. placebo, any AE by dose intensity by end of follow-up
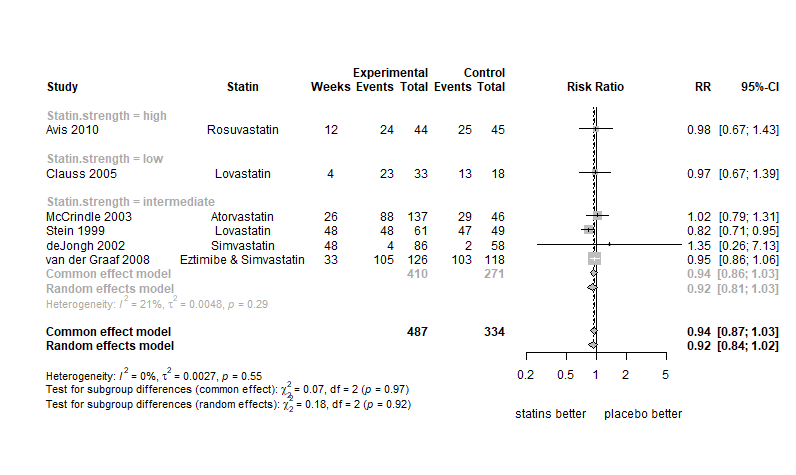


Supplementary Figure 16 Forest plot, statins vs. placebo, any serious adverse event by end of follow-up
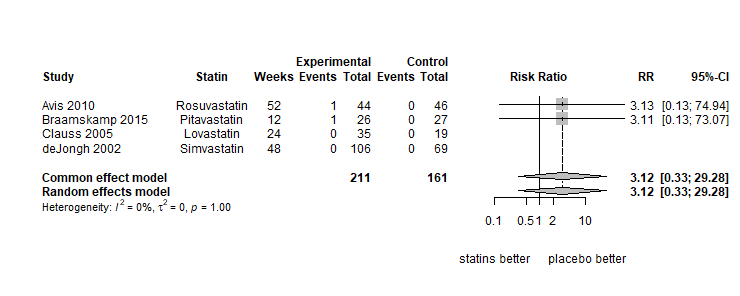


Supplementary Figure 17 Forest plot, statins vs. placebo, discontinuation due to adverse event by end of follow-up
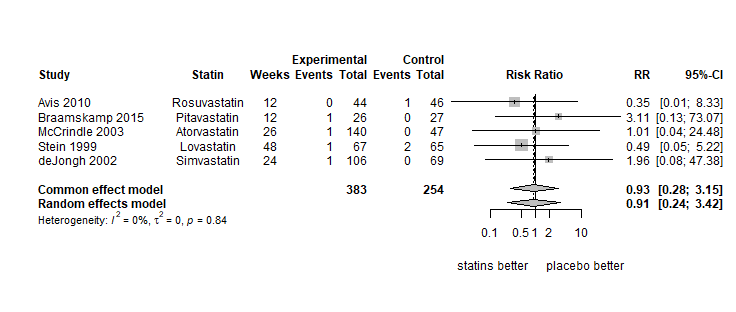


Supplementary Figure 18 Forest plot, statins vs. placebo, myalgia by end of follow-up


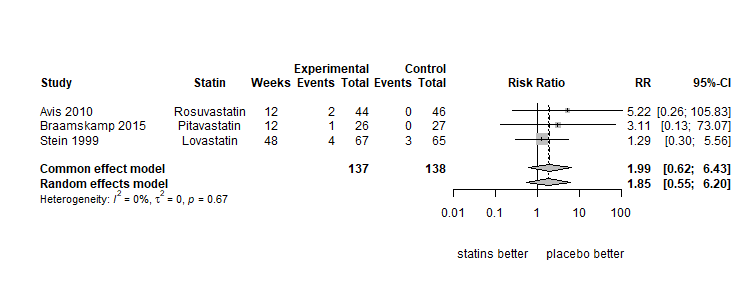


Figure 19 Contour-enhanced funnel plot, statins vs. placebo, % change from baseline in serum LDL-C


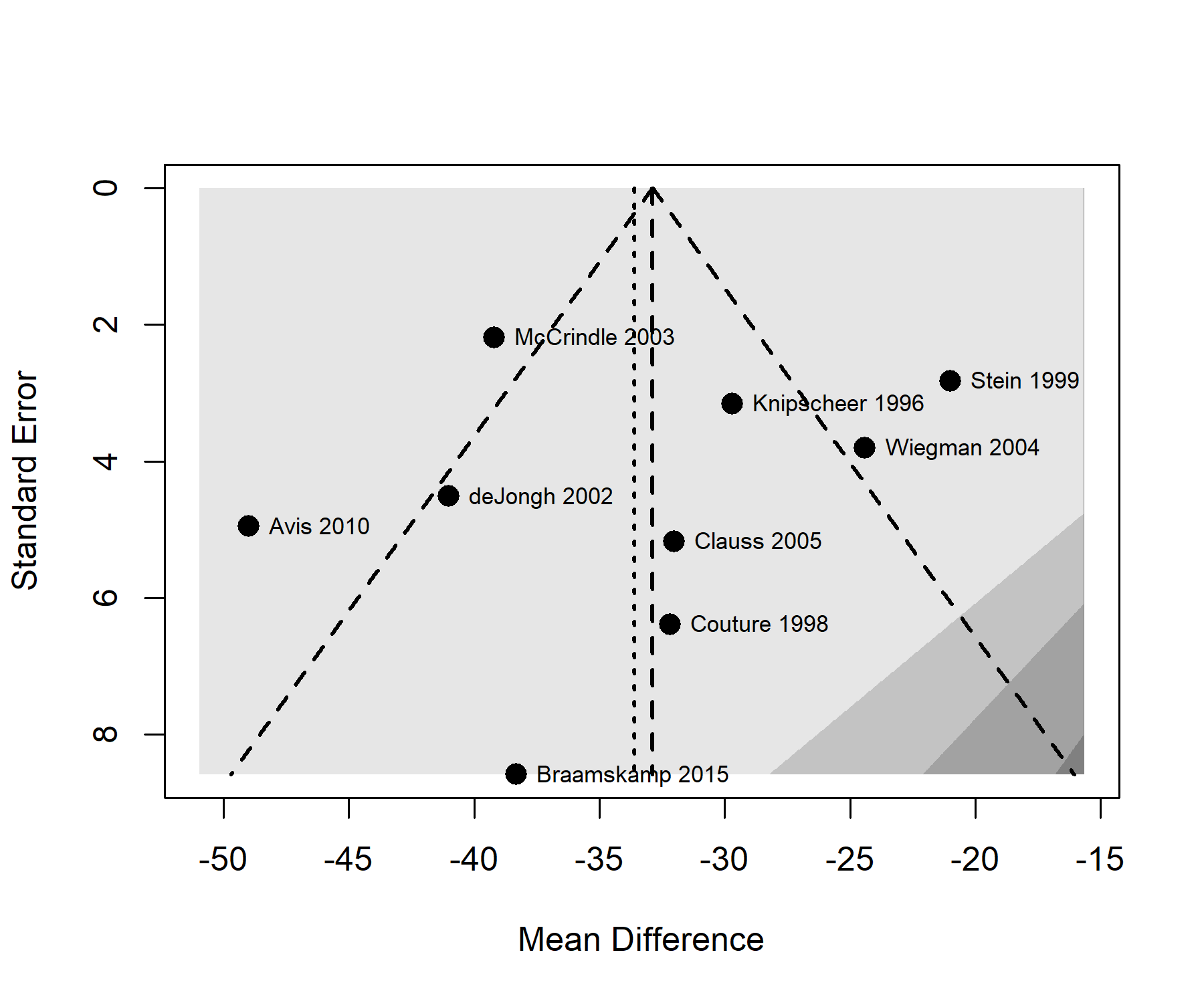

Supplement: Multimedia component 2 [file mmc2.docx]
