# Supplementary material for: Efficacy and safety of statins, ezetimibe and statins-ezetimibe therapies for children and adolescents with heterozygous familial hypercholesterolaemia: Systematic review, pairwise and network meta-analyses of randomised controlled trials
Source: Atherosclerosis. 2025 Feb;401:None. doi: 10.1016/j.atherosclerosis.2024.118598 (PMC11811749; doi:10.1016/j.atherosclerosis.2024.118598)
Supplement: Multimedia component 3 [file mmc3.docx]

# Supplementary tables

## Contents

[Supplementary tables 1](#_Toc171429441)

[Contents 1](#_Toc171429442)

[List of tables 1](#_Toc171429443)

[Diagnosis and selection criteria 2](#_Toc171429444)

[Risk of bias 4](#_Toc171429445)

[Additional results 5](#_Toc171429446)

[Pairwise comparisons, LDL-C, TC, HDL-C and TG outcomes at end of follow-up 5](#_Toc171429447)

[Pairwise comparisons, LDL-C target at end of follow-up 5](#_Toc171429448)

[NMA results 6](#_Toc171429449)

[Safety results 8](#_Toc171429450)

## List of tables

[Table 1 Trial diagnosis and selection criteria 2](#_Toc171429430)

[Table 2 Risk of bias assessment 4](#_Toc171429431)

[Table 3 Pairwise comparisons, LDL-C, TC, HDL-C and TG outcomes at end of follow-up 5](#_Toc171429432)

[Table 4 Pairwise comparisons, LDL-C target at end of follow-up 5](#_Toc171429433)

[Table 5 NMA results by drug class, LDL-C reduction from baseline at end of follow-up 6](#_Toc171429434)

[Table 6 Ranking of treatments by drug class, % LDL-C change from baseline at end of follow-up 6](#_Toc171429435)

[Table 7 NMA results by treatment (statins split by type), LDL reduction from baseline to end of follow-up 6](#_Toc171429436)

[Table 8 Ranking of treatments by drug type, %LDL change from baseline at end of follow-up 7](#_Toc171429437)

[Table 9 NMA results accounting for statin dose strength, %LDL change from baseline at end of follow-up 7](#_Toc171429438)

[Table 10 Ranking of treatments accounting for statin dose intensity, %LDL change from baseline at end of follow-up 8](#_Toc171429439)

[Table 11 Safety results summary 8](#_Toc171429440)

## Diagnosis and selection criteria

Table 1 Trial diagnosis and selection criteria

|  | **HeFH diagnosis criteria** | **Selection criteria** |
| --- | --- | --- |
| Avis 2010 | Genetic defect or NECP 1992 criteria; LDL-C ≧ 190 mg/dL or LDL-C > 160 mg/dL and early CVD in family or ≧2 other CVD risk factors | Inclusion: age 10 - 17 years, HeFH, Tanner stage ≧ II, females at least 1 year post-menarche |
| Braamskamp 2015 | Genetic testing (97%). LDL-C ≧ 160 mg/dL or LDL-C > 130 mg/dL and at least one of the following: male, early CVD in family, HDL-C < 45 mg/dL, TG > 150 mg/dL, lipoprotein(a) > 75 nmol/L, type 2 diabetes mellitus diagnosed and blood pressure > 95th percentile for age and height. | Inclusion: age 6 - 17 years, HeFH  Exclusion: NR |
| Clauss 2005 | 1 parent with FH, LDL-C 160-400mg/dL & TG<350mg/dL | Inclusion: age 10 - 17 years; female; LDL-C 4.1-10.3 mmol/L on diet; TG < 4.0 mmol/L; postmenarchal >1 year; 1 parent with FH* Exclusion: pregnancy; under/overweight; HoFH; dyslipidemia I, III-V; DM, hypothyroidism; renal disorder; certain medication (immunosuppressants, corticosteroids, cytochrome P-450 inhibitors)  Exclusion: NR |
| Couture 1998 | Genetic testing | Inclusion: age < 18 years; HeFH; LDL-C > 95th percentile on diet.  Exclusion: DM; anorexia; kidney, liver or thyroid disorder; delayed puberty. |
| de Jongh 2002 | 1 parent with clinical FH, LDL-C 4.1 to 10.3mmol/L | Inclusion: age ≦ 18 years; HeFH; LDL-C > 95th percentile; genetic diagnosis or family history of high LDL-C.  Exclusion: smoking; vasoactive medication; serious illness; HT; DM. |
| Harada-Shiba 2016 | JAC criteria for HeHF | Inclusion: age 10 - 17 years, HeFH, male, 10-15 years; LDL-C ≥190 mg/dL or LDL-C ≥160 mg/dL with family history of CAD, obesity, DM2 or hypertension; diet therapy for at least 3 months before screening. Exclusion: HoFH, secondary hyperlipidemia. |
| Knipscheer 1996 | HC in relative or early AS in family (1st-2nd degree), LDL-C > 95th percentile on diet; | Inclusion: age 8 - 16 years; HeFH ̃ LDL-C > 95th %tile on diet; HC or early AS in family.  Exclusion: major surgery within 3 months; drugs interfering with lipid metabolism; liver or renal dysfunction. |
| Kusters 2015 | LDL-C levels 189mg/dL to 400mg/dL and family history; or 159 to 400mg/dL and: genotyping, 1 parent with genotype HeFH and LDL-C>159mg/dL, 1 parent with >=210 mg/dL, or tendinous xanthomas not associated with other disorder | Inclusion: HeFH (90%) and clinically important nonFH (10%). TG>7.8 mmol/L, clinical laboratory values within normal limits or clinically acceptable to the investigator, alanine aminotransferase (ALT) and/or aspartate aminotransferase (AST) >=1.5 the upper limit of normal (ULN), serum creatinine <2.0 mg/dL (177 umol/L), and were free of any clinically important disease other than hypercholesterolemia.  Exclusion: hypersensitivity or any contraindication to ezetimibe; any cardiac disorder or disorders of the hematologic, digestive, or central nervous systems; uncontrolled endocrine metabolic disease; unstable thyroid hormone replacement therapy with thyroid stimulating hormone levels outside the normal range; impaired renal function/renal disease; hepatic or biliary disease; history of partial ileal bypass or disease that affects significant function of the ileum; HIV; coagulopathy; HoFH; LDL apheresis or plasma apheresis. Known lipid-altering therapies, foods, or supplements. |
| Lambert 1996 | HeFH diagnosis (severe). LDL-C > 95th centile on diet, family history of early AS, and family history of hyperlipidaemia with LDL-C >95th centile, or FH confirmed by molecular diagnosis | Inclusion: LDL-C >95th percentile for age while on diet, history of unsuccessful bile acid-binding treatment, positive FH of AS before 50, and a history of hyperlipidemia with LDL-C >95th percentile for age and sex or genetic diagnosis of FH.  Exclusion: Conditions such as diabetes, anorexia, kidney liver or thyroid disorder, ileal bypass, delayed puberty; weight or height < 3rd percentile or >97th percentile; serious illness or major surgery 3 mths prior; use of corticosteroids, anabolic steroieds, immunosuppressants, barbituates, anticonvulsants, anitcoagulants, other lipid lowering drugs, drug or alcohol abuse; type I-IV hyperlipidemia, HoFH, triglyceride > 4.0mmol/L. |
| McCrindle 2003 | FH or severe hypercholesterolemia and LDL-C >= 4.9 mmol/L OR LDL-C >= 4.1 mmol/L and family history of FH OR LDL-C > 4.1 mmol/L and premature CHD in 1°/2° relatives | Inclusion: age 10 - 17 years; HeFH or LDL-C ≧ 4.9 mmol/L or LDL-C ≧ 4.1 mmol/L with HC or early AS in family; Tanner ≧ II; LDL-C ≧ 4.1 mmol/L w/ diet during baseline phase.  Exclusion: premenarche; pregnancy; under or overweight; liver or kidney disorder; HoFH; other clini- cal trial; hypersensitivity to statins. |
| Stein 1999 | LDL-C > 4.9mmol/L and 1 parent LDL-C > 4.9mmol/l; or LDL-C > 5.7mmol/L and CAD death in 1 parent | Inclusion: age 10 - 17 years; LDL-C 4.9 - 13.0 mmol/L on diet and ≧ 1 parent with LDL-C ≧ 4.9 mmol/Lwith or LDL-C 5.7 - 13.0 mmol/L on diet and a parent died of CAD; (Tanner > I required later by FDA > 8participants needed to discontinue). Exclusion: delayed puberty; under/overweight; HoFH; secondary hyperlipidaemia; TG disorders. |
| Van der Graff 2008 | One of: 1) Genotype-confirmed HeFH and LDL-C 159 mg/dl and 400 mg/dl. 2) LDL-C 159 mg/dl and 400 mg/dl and at least 1 biological parent with genotype-confirmed HeFH and historical untreated LDL-C 159 mg/dl. 3) LDL-C 159 mg/dl and 400 mg/dl and at least 1 biological parent with untreated LDL-C of at least 210 mg/dl in the absence of another condition associated with secondary elevated LDL-C. 4) LDL-C 189 mg/dl and 400 mg/dl and a family history of hypercholesterolemia consistent with dominant autosomal transmission. 5) LDL-C 159 mg/dl and 400 mg/dl and tendinous xanthomas, without another condition associated with secondary elevated LDL-C | Inclusion: TG ≦350 mg/dl on diet, clinical laboratory values within normal limits, with baseline liver function tests and creatine phosphokinase 1.5 the upper limit of normal (ULN).  Exclusion: Any cardiac disorder including congenital cardiac disorders or hematologic, digestive, or central nervous system disorders; inadequately controlled or newly diagnosed diabetes mellitus; uncontrolled endocrine or metabolic disease known to influence serum lipids or lipoproteins; known impairment of renal function or other renal disease; active or chronic hepatobiliary or hepatic disease; human immunodeficiency virus; known coagulopathy; documented homozygous FH or laboratory values consistent with homozygous FH; use of LDL apheresis or plasma apheresis; partial ileal bypass; excessive alcohol use or drug abuse; delayed puberty. Documented mutations in the apolipoprotein B (apo B) gene in the absence of mutations in the LDL-C receptor gene. Relevant concomitant therapy or prior therapy within designated washout periods. |
| Wiegman 2004 | Molecular diagnosis in parent, LDL-C > 4.0 mmol/L and TG<350mg/dL | Inclusion: age 10 - 17 years; female; 1 parent with FH and LDL-C 4.1-10.3 mmol/L on diet; TG < 4.0mmol/L; postmenarchal > 1 year.  Exclusion: pregnancy; under/overweight; HoFH; dyslipidaemia I, III-V; DM, hypothyroidism; renal dis-order; certain medication (immunosuppressants, corticosteroids, cytochrome P-450 inhibitors). |

## Risk of bias

Table 2 Risk of bias assessment

| **Study ID** | **D1** | **D2** | **D3** | **D4** | **D5** | **Overall** |  |  |
| --- | --- | --- | --- | --- | --- | --- | --- | --- |
| Avis 2010 |  |  |  |  |  |  |  | Low risk |
| Braamskamp 2015 |  |  |  |  |  |  |  | Some concerns |
| Clauss 2005 |  |  |  |  |  |  |  | High risk |
| Couture 1998 |  |  |  |  |  |  |  |  |
| de Jongh 2002 |  |  |  |  |  |  | D1 | Randomisation process |
| Harada-Shiba 2016 |  |  |  |  |  |  | D2 | Deviations from the intended interventions |
| Knipscheer 1996 |  |  |  |  |  |  | D3 | Missing outcome data |
| Kusters 2015 |  |  |  |  |  |  | D4 | Measurement of the outcome |
| Lambert 1996 |  |  |  |  |  |  | D5 | Selection of the reported result |
| McCrindle 2003 |  |  |  |  |  |  |  |  |
| Stein 1999 |  |  |  |  |  |  |  |  |
| van der Graaf 2008 |  |  |  |  |  |  |  |  |
| Wiegman 2004 |  |  |  |  |  |  |  |  |

## Additional results

### Pairwise comparisons, LDL-C, TC, HDL-C and TG outcomes at end of follow-up

Table 3 Pairwise comparisons, LDL-C, TC, HDL-C and TG outcomes at end of follow-up

|  | **N studies (participants)** | **% change**  **(95% CI)*** | **N studies (participants)** | **Absolute change**  **mmol/L (95% CI)*** |
| --- | --- | --- | --- | --- |
| **Serum LDL-C** |  |  |  |  |
| Statins vs. pbo | 9 (933) | -33.61 (-39.63 to -27.58, I^2=^83%) | 9 (933) | -2.02 (-2.40 to -1.63, I^2=^69%)) |
| Ezetimibe vs. pbo | 1 (127) | -28.95 (-32.10 to -25.80) | 1 (127) | -1.63 (-2.22 to -1.04) |
| Sim+eze vs. sim+pbo | 1 (229) | -15.85 (-19.79 to -11.91) | 1 (229) | -0.97 (-1.36 to -0.58) |
| **Serum TC** |  |  |  |  |
| Statins vs. pbo | 8 (870) | -26.63 (-31.78 to -21.48, I^2^=90%) | 8 (750) | -2.00 (-2.44 to -1.56, I^2^=66%)) |
| Ezetimibe vs. pbo | 1 (127) | -21.20 (-25.11 to -17.29) | 1 (127) | -1.66 (-2.25 to -1.06) |
| Sim+eze vs. sim+pbo | 1 (229) | -13.20 (-13.50 to -12.90) | 1 (229) | -1.03 (-1.07 to -0.99) |
| **Serum HDL-C** |  |  |  |  |
| Statins vs. pbo | 8 (870) | 3.54 (1.00 to 6.08, I^2^=0%) | 8 (870) | 0.04 (-0.01 to 0.08, I^2^=0%))) |
| Ezetimibe vs. pbo | 1 (127) | 1.00 (-5.80 to 7.80) | 1 (127) | 0.00 (-0.14 to 0.14) |
| Sim+eze vs. sim+pbo | 1 (229) | 0.99 (0.66 to 1.32) | 1 (229) | 0.00 (-0.01 to 0.00) |
| **Serum TG** |  |  |  |  |
| Statins vs. pbo | 7 (659) | -7.97 (-12.87 to -3.07, I^2^=23%) | 5 (366) | -0.13 (-0.37 to 0.11, I^2^=45%) |
| Ezetimibe vs. pbo | 1 (127) | -14.00 ( -15.79 to -12.21) | 1 (127) | -0.26 (-0.95 to 0.43) |
| Sim+eze vs. sim+pbo | 1 (229) | median -6.96 (-15.35 to 1.43) | 1 (229) | median -0.28 (-0.68 to 0.11) |

* Expressed as mean % change unless otherwise specified

### Pairwise comparisons, LDL-C target at end of follow-up

Table 4 Pairwise comparisons, LDL-C target at end of follow-up

|  | **trials** | **% achieving serum LDL-C target** | |
| --- | --- | --- | --- |
|  |  | ***<3.4 mmol/L (<130 mg/dL)*** | ***<2.8 mmol/L***  ***(<110 mg/dL)*** |
| Atorvastatin vs. placebo | 1 | 60% vs. 0 (p<0.01) | NR |
| Rosuvastatin vs. placebo | 1 | NR | Ros. 5mg: 12%  10mg: 41%  20mg: 41%  Pbo: 0 |
| Simvastatin+ezetimibe vs. simvastatin+placebo | 1 | 77% vs. 53% (p<0.01) | 63% vs. 27% (p<0.01) |
| Ezetimibe vs. placebo | 0 | NA | NA |

### NMA results

#### NMA results by drug class

Table 5 NMA results by drug class, LDL-C reduction from baseline at end of follow-up

|  | **% change*** | | **mmol/L*** | | **mg/dL*** | |
| --- | --- | --- | --- | --- | --- | --- |
| **Comparison** | **MD** | **95% CrI** | **MD** | **95% CrI** | **MD** | **95% CrI** |
| Stat. vs. pbo | -33.46 | -40.99 to -26.06 | -1.99 | -2.52 to -1.49 | -77.02 | -2.52 to -1.49 |
| Eze vs. pbo | -28.88 | -49.72 to -7.65 | -1.63 | -3.11 to -0.18 | -62.95 | -3.11 to -0.18 |
| Eze+stat vs. pbo | -49.21 | -71.32 to -26.71 | -2.96 | -4.46 to-1.55 | -114.3 | -4.46 to -1.55 |
| Eze+stat vs. eze | -22.65 | -65.77 to 22.95 | -1.33 | -3.37 to 0.70 | -51.35 | -3.37 to 0.70 |
| Stat vs. eze | -4.58 | -26.64 to 17.68 | -0.36 | -1.89 to 1.20 | -14.08 | -1.89 to 1.20 |
| Stat vs. stat+eze | 15.75 | -5.31 to 37.11 | 0.96 | -0.33 to 2.33 | 37.27 | -0.33 to 2.33 |

*Negative values favour the first treatment in the comparison, and positive values favour the second.

Results highlighted in grey scale are not statistically significant at conventional levels.

Table 6 Ranking of treatments by drug class, % LDL-C change from baseline at end of follow-up

| **Treatment** | **Rank** | **Ranking**  **probability** | **Mean**  **rank** | **95% CrI** |
| --- | --- | --- | --- | --- |
| Statins+ezetimibe | 1 | 90% | 1.1 | 1 to 3 |
| Statins | 2 | 66% | 2.3 | 1 to 3 |
| Ezetimibe | 3 | 66% | 2.6 | 1 to 3 |
| Placebo | 4 | 99% | 4.0 | 4 to 4 |

Ranking probability is defined as the probability that a treatment out of n treatments in an NMA is the best, the second, the third, and so on until the least effective treatment.

#### NMA results by treatment

Table 7 NMA results by treatment (statins split by type), LDL reduction from baseline to end of follow-up

|  | **% change*** | | **mmol/L*** | | **mg/dL*** | |
| --- | --- | --- | --- | --- | --- | --- |
| **Comparison** | **MD** | **95% CrI** | **MD** | **95% CrI** | **MD** | **95% CrI** |
| Ato vs. Pbo | -38.84% | -64.99 to -9.14 | -2.20 | -3.82 to -0.55 | -85.06 | -147.88 to -21.44 |
| Eze vs. Pbo | -28.50% | -54.07 to -1.52 | -1.63 | -3.49 to 0.08 | -62.90 | -134.92 to 3.27 |
| Eze & Sim vs. Pbo | -51.15% | -82.57 to -17.85 | -2.85 | -4.60 to -0.55 | -110.19 | -177.96 to-21.09 |
| Lov vs. Pbo | -25.35% | -45.72 to -6.54 | -1.54 | -2.74 to -0.31 | -59.55 | -106.08 to -11.92 |
| Pita vs. Pbo | -38.05% | -67.24 to -6.54 | -2.45 | -4.37 to -0.33 | -94.66 | -169.07 to -12.74 |
| Prav vs. Pbo | -26.72% | -44.10 to -6.62 | -1.52 | -3.00 to -0.30 | -58.67 | -116.10 to -11.48 |
| Ros vs. Pbo | -49.38% | -78.60 to -21.52 | -3.05 | -4.82 to -1.44 | -118.13 | -186.21 to -55.73 |
| Sim vs. Pbo | -35.61% | -55.48 to -15.33 | -1.93 | -2.92 to -0.77 | -74.58 | -112.95 to -29.78 |
| Eze vs. Ato | 10.33% | -29.34 to 47.65 | 0.57 | -2.16 to 2.77 | 22.16 | -83.63 to 107.30 |
| Eze & Sim vs. Ato | -12.31% | -56.60 to 30.72 | -0.65 | -2.92 to 1.86 | -25.13 | -112.80 to 71.93 |
| Lov vs. Ato | 13.49% | -22.29 to 46.20 | 0.66 | -1.51 to 2.68 | 25.51 | -58.57 to 103.69 |
| Pita vs. Ato | 0.79% | -39.81 to 39.41 | -0.25 | -2.64 to 2.25 | -9.60 | -102.16 to 86.88 |
| Prav vs. Ato | 12.12% | -19.52 to 45.35 | 0.68 | -1.65 to 2.69 | 26.38 | -63.73 to 103.91 |
| Ros vs. Ato | -10.54% | -52.00 to 27.19 | -0.86 | -3.27 to 1.49 | -33.07 | -126.63 to 57.46 |
| Sim vs. Ato | 3.23% | -32.02 to 36.74 | 0.27 | -1.50 to 2.20 | 10.48 | -57.91 to 85.12 |
| Eze & Sim vs. Eze | -22.65% | -65.77 to 22.95 | -1.22 | -3.66 to 1.82 | -47.29 | -141.52 to 70.31 |
| Lov vs. Eze | 3.16% | -32.07 to 34.27 | 0.09 | -1.98 to 2.39 | 3.35 | -76.39 to 92.58 |
| Pita vs. Eze | -9.54% | -48.57 to 30.06 | -0.82 | -3.36 to 1.63 | -31.76 | -130.12 to 63.19 |
| Prav vs. Eze | 1.79% | -31.04 to 35.71 | 0.11 | -2.06 to 2.51 | 4.23 | -79.77 to 97.15 |
| Ros vs. Eze | -20.87% | -61.57 to 16.20 | -1.43 | -3.76 to 1.24 | -55.23 | -145.32 to 47.94 |
| Sim vs. Eze | -7.10% | -41.51 to 26.14 | -0.30 | -2.15 to 1.98 | -11.68 | -83.27 to 76.74 |
| Lov vs. Eze & Sim | 25.80% | -13.83 to 63.68 | 1.31 | -1.16 to 3.33 | 50.64 | -44.91 to 128.89 |
| Pita vs. Eze & Sim | 13.10% | -30.97 to 56.56 | 0.40 | -2.96 to 3.14 | 15.53 | -114.31 to 121.55 |
| Prav vs. Eze & Sim | 24.43% | -13.19 to 63.08 | 1.33 | -1.48 to 3.38 | 51.52 | -57.22 to 130.64 |
| Ros vs. Eze & Sim | 1.77% | -41.00 to 41.17 | -0.21 | -3.06 to 2.17 | -7.93 | -118.32 to 83.93 |
| Sim vs. Eze & Sim | 15.54% | -11.07 to 40.94 | 0.92 | -0.93 to 2.42 | 35.61 | -35.87 to 93.63 |
| Pita vs. Lov | -12.70% | -47.72 to 40.94 | -0.91 | -3.38 to 1.61 | -35.11 | -130.82 to 62.22 |
| Prav vs. Lov | -1.37% | -26.23 to 27.57 | 0.02 | -1.75 to 1.74 | 0.88 | -67.80 to 67.38 |
| Ros vs. Lov | -24.03% | -58.48 to 11.38 | -1.51 | -3.64 to 0.66 | -58.57 | -140.92 to 25.35 |
| Sim vs. Lov | -10.26% | -37.49 to 20.01 | -0.39 | -2.01 to 1.33 | -15.03 | -77.69 to 51.57 |
| Prav vs. Pita | 11.33% | -24.58 to 47.31 | 0.93 | -1.92 to 3.38 | 35.99 | -74.06 to 130.59 |
| Ros vs. Pita | -11.33% | -53.37 to 28.65 | -0.61 | -3.15 to 1.88 | -23.46 | -121.66 to 72.61 |
| Sim vs. Pita | 2.44% | -33.30 to 35.95 | 0.52 | -1.70 to 2.84 | 20.08 | -65.66 to 109.77 |
| Ros vs. Prav | -22.66% | -57.88 to 10.58 | -1.54 | -3.61 to 0.78 | -59.45 | -139.58 to 30.18 |
| Sim vs. Prav | -8.89% | -37.10 to 17.40 | -0.41 | -1.99 to 1.45 | -15.91 | -76.76 to 56.01 |
| Sim vs. Ros | 13.77% | -18.97 to 49.96 | 1.13 | -0.77 to 3.33 | 43.55 | -29.58 to 128.61 |

* Negative values favour the first treatment in the comparison, and positive values favour the second.

Results highlighted in grey scale are not statistically significant at conventional levels.

Table 8 Ranking of treatments by drug type (statins split by type), %LDL change from baseline at end of follow-up

| **Treatment** | **Rank** | **Ranking**  **probability** | **Mean**  **rank** | **95% CrI** |
| --- | --- | --- | --- | --- |
| Ezetimibe & Simvastatin | 1 | 56% | 1.8 | 1 to 6 |
| Rosuvastatin | 2 | 39% | 2.4 | 1 to 6 |
| Atorvastatin | 3 | 27% | 3.9 | 1 to 8 |
| Pitavastatin | 4 | 16% | 4.2 | 1 to 8 |
| Simvastatin | 4 | 29% | 4.3 | 2 to 7 |
| Ezetimibe | 6 | 30% | 6.0 | 2 to 8 |
| Pravastatin | 7 | 32% | 6.4 | 3 to 8 |
| Lovastatin | 8 | 43% | 6.9 | 3 to 8 |
| Placebo | 9 | 96% | 8.9 | 8 to 9 |

Ranking probability is defined as the probability that a treatment out of n treatments in an NMA is the best, the second, the third, and so on until the least effective treatment.

#### NMA results by dose strength

Table 9 NMA results accounting for statin dose strength, %LDL change from baseline at end of follow-up

|  | **% change*** | | **mmol/L*** | | **mg/dL*** | |
| --- | --- | --- | --- | --- | --- | --- |
| **Comparison** | **MD** | **95% CrI** | **MD** | **95% CrI** | **MD** | 95% CrI |
| Eze vs. Pbo | -29.00 | -39.99 to -18.01 | -1.63 | -2.31 to -0.93 | -63.01 | -89.29 to -35.80 |
| Eze & Stat^#^ vs. Pbo | -42.84 | -55.71 to -29.94 | -2.48 | -3.13 to -1.87 | -95.77 | -121.20 to -72.20 |
| High vs. Pbo | -42.33 | -51.25 to -34.11 | -2.53 | -3.03 to -2.04 | -97.85 | -117.13 to -78.92 |
| Low vs. Pbo | -27.01 | -32.11 to -21.90 | -1.50 | -1.80 to -1.22 | -58.00 | -69.72 to -47.15 |
| Med vs. Pbo | -31.78 | -37.42 to -26.55 | -1.90 | -2.21 to -1.61 | -73.58 | -85.33 to -62.37 |
| Eze & Stat^#^ vs. Eze | -13.84 | -30.39 to 2.67 | -0.85 | -1.80 to 0.05 | -32.77 | -69.54 to 1.99 |
| High vs. Eze | -13.33 | -27.71 to -0.19 | -0.90 | -1.78 to -0.07 | -34.84 | -68.81 to -2.78 |
| Low vs. Eze | 1.99 | -10.16 to 14.03 | 0.13 | -0.62 to 0.86 | 5.00 | -24.13 to 33.35 |
| Med vs. Eze | -2.78 | -15.53 to 8.92 | -0.27 | -1.04 to 0.45 | -10.57 | -40.26 to 17.59 |
| High vs. Eze & Stat^#^ | 0.51 | -15.03 to 15.49 | -0.05 | -0.82 to 0.75 | -2.08 | -31.68 to 29.03 |
| Low vs. Eze & Stat^#^ | 15.83 | 4.26 to 27.76 | 0.98 | 0.42 to 1.55 | 37.77 | 16.32 to 60.12 |
| Med vs. Eze & Stat^#^ | 11.06 | -2.51 to 24.19 | 0.57 | -0.05 to 1.24 | 22.20 | -2.06 to 47.85 |
| Low vs. High | 15.32 | 6.01 to 25.36 | 1.03 | 0.46 to 1.60 | 39.84 | 17.97 to 61.98 |
| Med vs. High | 10.55 | 1.62 to 19.80 | 0.63 | 0.09 to 1.16 | 24.27 | 3.53 to 44.70 |
| Med vs. Low | -4.77 | -11.22 to 1.05 | -0.40 | -0.73 to -0.08 | -15.57 | -28.21 to -2.95 |

*Negative values favour the first treatment in the comparison, and positive values favour the second.

^#^ Intermediate dose statins combined with ezetimibe.

Results highlighted in grey scale are not statistically significant at conventional levels.

Eze: Ezetimibe; Pbo: placebo; Med: Medium/intermediate intensity statins; Stat: statin

Table 10 Ranking of treatments accounting for statin dose intensity, %LDL change from baseline at end of follow-up

|  | **Rank** | **Ranking**  **probability** | **Mean rank** | **95% CrI** |
| --- | --- | --- | --- | --- |
| Statins (higher) | 1 | 66% | 1.4 | 1 to 3 |
| Ezetimibe & Simvastatin | 2 | 57% | 1.8 | 1 to 4 |
| Statins (intermediate) | 3 | 72% | 3.1 | 2 to 4 |
| Ezetimibe | 5 | 41% | 4.1 | 2 to 5 |
| Satins (lower) | 5 | 57% | 4.5 | 4 to 5 |
| Placebo | 6 | 100% | 6.0 | 6 to 6 |

### Safety results

Table 11 Safety results summary

|  | **N studies** | **N**  **participants** | **Results^*^** |
| --- | --- | --- | --- |
| **Maturation^#^** |  |  |  |
| Statins vs. pbo | 3 | 576 | 0.91 (0.75 to 1.11, I^2^=0%) |
| Ezetimibe vs. pbo | 0 | NA | NA |
| Sim+eze vs. sim+pbo | 0 | NA | NA |
| **Adverse events (all)** |  |  |  |
| Statins vs. pbo | 5 | 638 | 1.00 (0.87 to 1.16, I^2^=0%) |
| Ezetimibe vs. pbo | 0 | NA | NA |
| Sim+eze vs. sim+pbo | 1 | 248 | RR 0.99 (0.89 to 1.10) |
| **Serious adverse events** |  |  |  |
| Statins vs. pbo | 4 | 372 | 3.12 (0.33 to 29.28, I^2^=0%) |
| Ezetimibe vs. pbo | 1 | 138 | 2/93 vs. 0/45 |
| Sim+eze vs. sim+pbo | 0 | NA | NA |
| **AE discontinuation** |  |  |  |
| Statins vs. pbo | 5 | 637 | 0.91 (0.24 to 3.42, I^2^=0%) |
| Ezetimibe vs. pbo | 1 | 138 | 3 vs. 0 events. |
| Sim+eze vs. sim+pbo | 0 | NA | NA |
| **Myopathy^+^** |  |  |  |
| Statins vs. pbo | 4 | 450 | 3/243 vs. 1/207 |
| Ezetimibe vs. pbo | 1 | 138 | 0/93 vs. 0/45 |
| Sim+eze vs. sim+pbo | 1 | 248 | 2/126 vs. 0/122 |
| **Liver dysfunction^^^** |  |  |  |
| Statins vs. pbo | 8 | 940 | 4/560 vs. 2/380 |
| Ezetimibe vs. pbo | 1 | 138 | 1/93 vs. 0/45 |
| Sim+eze vs. sim+pbo | 1 | 248 | 6/126 vs. 3/122 |
| **Rhabdomyolysis** |  |  |  |
| Statins vs. pbo | 1 | 44 | 0 events |
| Ezetimibe vs. pbo | 1 | 138 | 0 events |
| Sim+eze vs. sim+pbo | 0 | NA | NA |
| **Myalgia** |  |  |  |
| Statins vs. pbo | 3 | 275 | 1.99 (0.62 to 6.43, I^2^=0%)) |
| Ezetimibe vs. pbo | 1 | 138 | 0 events |
| Sim+eze vs. sim+pbo | 1 | 248 | 7 vs 1 events |

* Results are reported either as relative risks (95% CI), or as n of events were data were too sparse for meta-analysis. Pooled estimates are from fixed-effect meta-analyses. Values below 1 favour statins, values above 1 favour placebo.

^#^ Presented as the relative risk of progressing by a Tanner stage of 1 or more within the follow-up period.

^+^ Defined as creatine kinase (CK) elevation >x10 the upper limit of normal (ULN) or as reported in the paper.

^ Clinically significant increase in liver transaminase values, defined as over three-fold increase in alanine transferase or aspartate aminotransferase.
